# METTL3 enhances pancreatic ductal adenocarcinoma progression and gemcitabine resistance through modifying *DDX23* mRNA N6 adenosine methylation

**DOI:** 10.1038/s41419-023-05715-1

**Published:** 2023-03-28

**Authors:** Chengjie Lin, Ting Li, Yan Wang, Shihui Lai, Yue Huang, Zhenyun Guo, Xiang Zhang, Shangeng Weng

**Affiliations:** 1 Department of Hepatopancreatobiliary Surgery, The First Affiliated Hospital of Fujian Medical University, Fuzhou, Fujian 350001 China; 2 Fujian Abdominal Surgery Research Institute, The First Affiliated Hospital, Fujian Medical University, Fuzhou, Fujian 350001 China; 3 National Regional Medical Center, Binhai Campus of the First Affiliated Hospital, Fujian Medical University, Fuzhou, Fujian 350212 China; 4 Department of Oncology, Fujian Provincial Hospital, Provincial Clinical College of Fujian Medical University, Fuzhou, Fujian 350001 China

**Keywords:** Cancer genetics, Cell death

## Abstract

The aim of the present study was to clarify the mechanism of how METTL3 regulated pancreatic ductal adenocarcinoma (PDAC) progression by m6A modification of its downstream target mRNA and signaling pathway. Immunoblotting and qRT-PCR assays was employed to determine the expression levels of METTL3. In situ fluorescence hybridization was conducted to localize the cellular distribution of METTL3 and DEAD-box helicase 23 (DDX23). CCK8, colony formation, EDU incorporation, TUNEL, wound healing and Transwell assays were carried out accordingly to study the viability, proliferation, apoptosis, and mobility of cells under different treatments in vitro. Xenograft and animal lung metastasis experiments were also conducted to study the functional role of METTL3 or DDX23 on tumor growth and lung metastasis in vivo. MeRIP-qPCR and bioinformatical analyses were used to obtain the potential direct targets of METTL3. It was shown that m6A methyltransferase METTL3 was upregulated in PDAC tissues with gemcitabine resistance, and its knockdown sensitized pancreatic cancer cells to chemotherapy. Furthermore, silencing METTL3 remarkably reduced pancreatic cancer cell proliferation, migration, and invasion both in vitro and in vivo. Mechanistically, validation experiments confirmed that *DDX23* mRNA was a direct target of METTL3 in YTHDF1-dependent manner. Additionally, DDX23 silence resulted in the suppression of pancreatic cancer cell malignancy and PIAK/Akt signaling inactivation. Strikingly, rescuse experiments demonstrated the inhibitive effects of METTL3 silence on cell phenotypes and gemcitabine resistance were partially reversed by forcibly expressed DDX23. In summary, METTL3 promotes PDAC progression and gemcitabine resistance by modifying DDX23 mRNA m6A methylation and enhancing PI3K/Akt signaling activation. Our findings establish a potential tumor promotive and chemo-resistant role for METTL3/DDX23 axis in PDAC.

## Background

Pancreatic ductal adenocarcinoma (PDAC) has been shown to demonstrate the worst survival outcome among all human cancers based on the statistics of the American Cancer Society. According to cancer statistics 2021, PDAC is with a 5-year survival rate of 10% [1]. Early diagnosis of pancreatic cancer is also very difficult because of the lack of specific symptoms [2]. Gemcitabine (GEM) has been frequently used to treat patients with pancreatic cancer, with either local invasion or distant metastasis [3]. Gemcitabine is a deoxycytidine nucleotide analog and is used as a standard first-line chemotherapeutic agent for pancreatic cancer. Unfortunately, patients frequently obtained resistance to GEM, resulting in a high rate of mortality [4]. And those with GEM resistance and metastasis demonstrated a 5-year survival rate lower than 5% [5]. Thus, the lack of early diagnostic biomarkers and symptoms as well as frequently occurring GEM resistance are critical issues that need to be addressed in effective pancreatic cancer treatment. As a result, efforts need to be put into the identification of potential early-diagnosis biomarkers and of mechanism that helps address GEM resistance.

N6-methyladenosine (m6A) modification is the most abundant form of posttranscriptional RNA modification in eukaryotes. Functions of m6A modification are implemented by RNA methyltransferases, RNA demethylase, and m6A binding proteins [6]. As an RNA m6A methyltransferase that catalyzes m6A, METTL3 was reported by Li et al. to be upregulated and promote oxaliplatin (the first-line treatment for advanced gastric cancer) resistance of CD133 + stem cells by enhancing PARP1 mRNA stability in gastric cancer [7]. METTL3 was also found upregulated and oncogenic in pancreatic cancer, demonstrated by promoting cell proliferation and invasion [8]. In addition, METTL3 was also reported to promote resistance to chemotherapy, including GEM, 5-fluorouracil, cisplatin, and radiotherapy of pancreatic cancer cells [9]. Despite the increased number of published studies regarding the role of m6A modification mediated by METTL3 in cancer, it is still of great need to obtain a deeper insight into the role of METTL3 in pancreatic cancer.

The DEAD-box helicase 23 (DDX23) gene encodes a member of the DEAD-box protein family. DEAD-box proteins are putative RNA helicases and are characterized by the conserved motif Asp-Glu-Ala-Asp (DEAD). DDX23 protein is a component of the U5 snRNP (small uridine-rich ribonucleoprotein) complex. Zhao et al. reported that DDX23, as a splicing factor, could be transcriptionally activated by E2F1, thus promoting ovarian cancer progression by regulating FOXM1 [10]. And recurrent somatic mutations or expression dysregulation of several U5 snRNP proteins, including DDX23 were reported to be associated with human cancers [11]. So far, how DDX23 is regulated and participates in cancer genesis has remained largely unknown.

This research was conducted to study the underlying mechanism of m6A’s pathological relevance to PDAC mediated by METTL3. The m6A writer METTL3 was found to be upregulated in gemcitabine-resistant PDAC samples and METTL3 silence sensitized pancreatic cancer cells to chemotherapy. Additionally, METTL3 knockdown inhibited PDAC aggressive tumor phenotypes through, at least in part, by weakening *DDX23* mRNA m6A modification. Therefore, METTL3/DDX23 axis might be a promising target to reverse gemcitabine resistance of PDAC.

## Results

### METTL3 was upregulated in GEM-resistant pancreatic cancer and its knockdown suppressed cancer progression

**Fig. 1:**
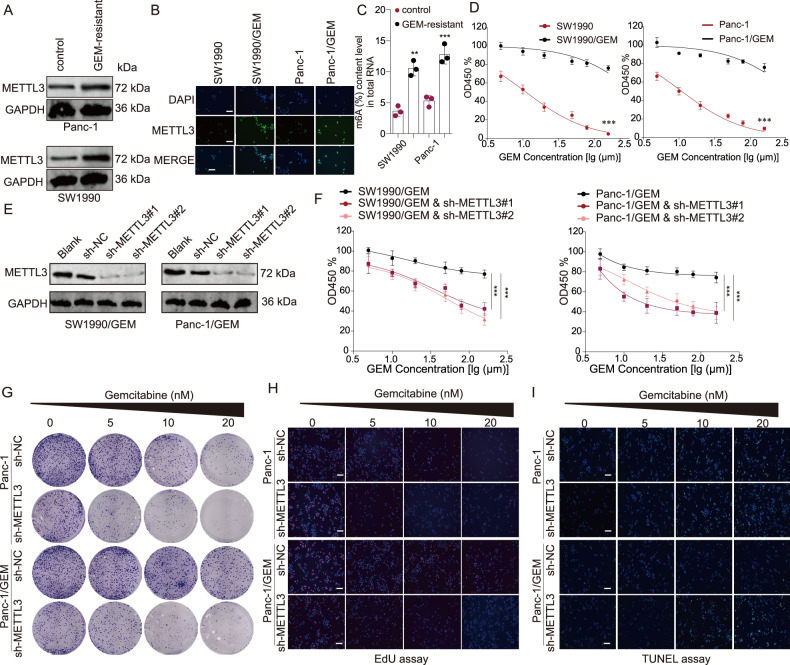
METTL3 was upregulated in gemcitabine-resistant cells and the knockdown of METTL3 increased the sensitivity to gemcitabine in pancreatic cells. **A**, **B**. METTL3 was upregulated in GEM-resistant Panc-1 and SW1990 cells. GEM: gemcitabine. **A** western blotting assay; **B** fluorescence in situ hybridization (FISH) assay. **C** The m6A level in GEM-resistant Panc-1 and SW1990 cells was significantly higher than in corresponding parent cells. **P < 0.01, ***P < 0.001, compared with control group. **D** Both SW1990 and Panc-1 cell lines were sensitive to GEM treatment in a dose-dependent manner whereas GEM-resistant cell lines did not show significant changes across the spectrum of doses of gemcitabine. ***P < 0.001, compared with the corresponding parent cell line. **E** The two shRNAs against METTL3 inhibited the protein expression of METTL3 in both GEM-resistant cell lines. ***P < 0.001, compared with the GEM-resistant cell line. **F** The knockdown of METTL3 in GEM-resistant cells significantly increased the sensitivity to gemcitabine treatment and resulted in less cell viability (measured in optical density (OD) at 450 nm). **G** In both Panc-1 and GEM-resistant Panc-1 cell lines, the knockdown of METTL3 significantly increased the sensitivity to gemcitabine treatment and led to less colony formation. **H** In both Panc-1 and GEM-resistant Panc-1 cell lines, the knockdown of METTL3 significantly increased the sensitivity to gemcitabine treatment and led to less EDU incorporation. **I** In both Panc-1 and GEM-resistant Panc-1 cell lines, the knockdown of METTL3 significantly increased the sensitivity to gemcitabine treatment and led to more cell apoptosis.

**Fig. 2:**
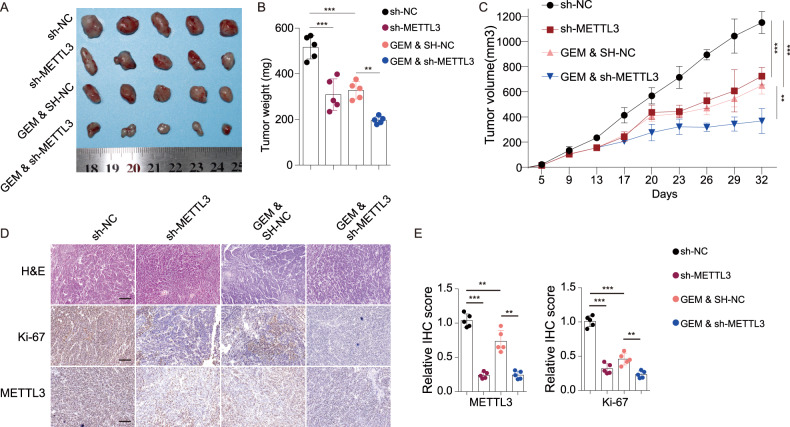
METTL3 knockdown suppressed in vivo tumor growth in synergy with gemcitabine treatment. **A** A photograph of tumors from xenograft mice models with different treatments. **B**, **C** The weight (**B**) and volume (**C**) of tumors of xenograft mice models in the four groups. **D** Representative H&E, Ki67 IHC, and METTL3 IHC results of tumor tissues of xenograft mice models. **E** The statistical analyses of METTL3 and Ki67 IHC results. sh-NC sh-METTL3 negative control, GEM gemcitabine treatment. For **B**, **C**, **E** **P < 0.01, ***P < 0.001, compared with the sh-NC group.

A previous study indicated that METTL3 was upregulated in pancreatic cancer tissues compared with the cancer-adjacent tissues [12]. We herein found that METTL3, which located in nuclei, was upregulated in GEM-resistant SW1990 and Panc-1 cell lines (Fig. 1A, B). Evidence showed that the total RNA m6A level in GEM-resistant cells was much higher than in the parent cells (Fig. 1C), and GEM resistance allowed cells to have a much higher IC_50_ than the sensitive cells (Fig. 1D). To reveal the biological role of METTL3 in PDAC, METTL3 silencing were performed with shRNAs in the two GEM-resistant cell lines (Fig. 1E). The results of the CCK8 assay, colony formation assay and EDU incorporation assay demonstrated that METTL3 knockdown led to suppressed cell proliferation in both GEM-resistant Panc-1 cell line and the parent cell lines (Fig. 1F–H). In addition, METTL3 depletion resulted in more cell apoptosis in the Panc-1 cell line and GEM-resistant Panc-1 cell line (Fig. 1I). Notably, we observed that METTL3 silence effectively suppressed tumor growth in both the subcutaneous parent cell line and GEM-resistant cell line transplantation models in nude mice (Fig. 2A–C). Moreover, the H&E pathological and Ki67 IHC staining revealed that mice implanted with either GEM-sensitive cells or GEM-resistant cells demonstrated significantly suppressed tumor aggression following METTL3 knockdown (Fig. 2D, E). More importantly, METTL3 silencing have no statistical effect on cell proliferation of normal pancreas ductal epithelial cells HPDE6-C7 (Fig. S1). Taking together, our results suggest that METTL3 promotes GEM-resistant and malignant biological behaviors of PDAC cells.

### DDX23 was identified as a downstream target of METTL3

**Fig. 3:**
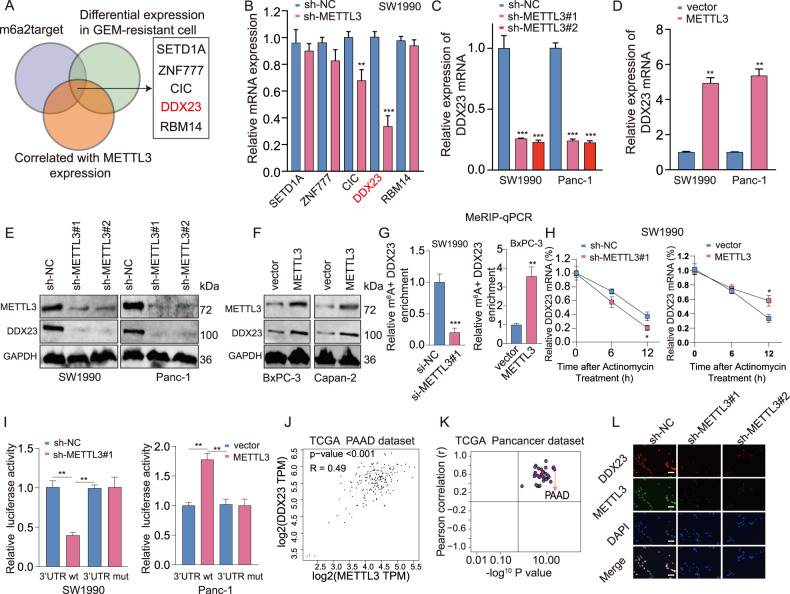
The identification and validation of a direct downstream target of METTL3 in pancreatic cancer under gemcitabine resistance. **A** The intersection between the list of predicted m6A targets of METTL3 by m6A2target, the list of differentially expressed genes in GEM-resistant pancreatic cancer, and the list that contained genes correlated with METTL3 expression showed the potential direct downstream targets of METTL3 in pancreatic cancer. **B** The relative mRNA expression of the identified five genes: SETD1A, ZNF777, CIC, DDX23, and RBM14 in cells with METTL3 silence. **P < 0.01, ***P < 0.001, compared with the sh-NC group. **C**, **D** The expression of DDX23 mRNA in the two cell lines with METTL3 knockdown (**C**) and METTL3 overexpression (**D**) was analyzed by qRT-PCR. **C** ***P < 0.001, compared with the sh-NC group. **D** **P < 0.01, compared with the vector group. **E**, **F** The expression of DDX23 protein in the two cell lines with METTL3 knockdown (**E**) and METTL3 overexpression (**F**) was determined using western blotting. **G** The m6A level of DDX23 in pancreatic cell lines with METTL3 knockdown (left panel) and overexpression (right panel) was measured. ***P < 0.001, compared with the sh-NC group; **P < 0.01, compared with the vector group. **H** The half-life of DDX23 mRNA in cells with respective METTL3 knockdown and overexpression with Actinomycin treatment was analyzed using qRT-PCR. **I** The regulatory relationship between METTL3 and DDX23 mRNA was validated using the dual-luciferase reporter gene assay. **J** In TCGA pancreatic cancer data, METTL3 and DDX23 were found significantly positively correlated. PAAD: pancreatic adenocarcinoma. **K** In all the cancers of TCGA, METTL3 expression was found significantly correlated with DDX23 expression. **L** The FISH assay showed that METTL3 knockdown led to DDX23 downregulation in the pancreatic cancer cell line.

To gain mechanistic insights into the regulatory role of METTL3 in PDAC, we pre-screened the candidate downstream target mRNAs of METTL3 using m6atarget online tool (http://rm2target.canceromics.org), the differentially expressed genes in GEM-resistant cells, and genes that were correlated with METTL3 expression. We identified five candidate genes: SETD1A, ZNF777, CIC, DDX23, and RMB14 (Fig. 3A), and further evaluated the expression of these five genes in METTL3 depleted SW1990 cells. Among these targets, DDX23 mRNA level decreased most significantly upon METTL3 depletion (Fig. 3B). METTL3 knockdown resulted in more than 70% reduction of DDX23 mRNA expression (Fig. 3C), whereas METTL3 overexpression led to over 5-fold upregulation of DDX23 mRNA (Fig. 3D). In addtion, similar results were observed in the protein expression (Fig. 3E, F). Mechanistically, to confirm whether DDX23 mRNA undergoes METTL3-mediated m6A modification, we performed methylated RNA immunoprecipitation quantitative PCR (meRIP-qPCR). Results showed that m6A level of DDX23 mRNA was obviously decreased upon METTL3 silencing, while it was increased following METTL3 upregulation (Fig. 3G). These findings suggested that METTL3 could methylate DDX23 mRNA.

Importantly, METTL3 silencing led to a significant decrease in the DDX23 transcript half-life after treatment with the transcriptional inhibitor actinomycin D, whereas opposite result was obtained after overexpressing METTL3 (Fig. 3H). Moreover, increased or decreased luciferase activity of constructs harboring the wild type DDX23 mRNA 3′-UTR were observed following METTL3 upregulation or downregulation, respectively. Conversely, significant results were obtained with the mutated DDX23 mRNA 3′-UTR sequence (Fig. 3I). Notably, METTL3 and DDX23 expression showed a significantly positive correlation (Fig. 3J). Besides, in all the 32 cancer types of the TCGA database, METTL3 and DDX23 demonstrated significantly positive correlations (Fig. 3K). Lastly, by fluorescence staining, we confirmed that METTL3 downregulation led to significantly DDX23 downregulation in nuclei (Fig. 3L). More importantly, DDX23 expression level in METTL3-Mutant cells was no different from controls group, indicating that METTL3 regulate DDX23 through distinct mechanisms depending on METTL3’s catalytic activity (Fig. S2). Interestingly, ectopic m6A “reader” protein YTHDF1 expression, not YTHDF2 markedly enhanced DDX23 expression (Fig. S3A). Meanwhile, YTHDF1 silencing obviously abolished the METTL3 mediated positive regulational effects on DDX23 expression (Fig. S3B). Together, these results document the regulation of *DDX23* mRNA under METTL3 in an m6A-dependent manner.

### DDX23 was found significantly upregulated in PDAC and predicted poor survival

**Fig. 4:**
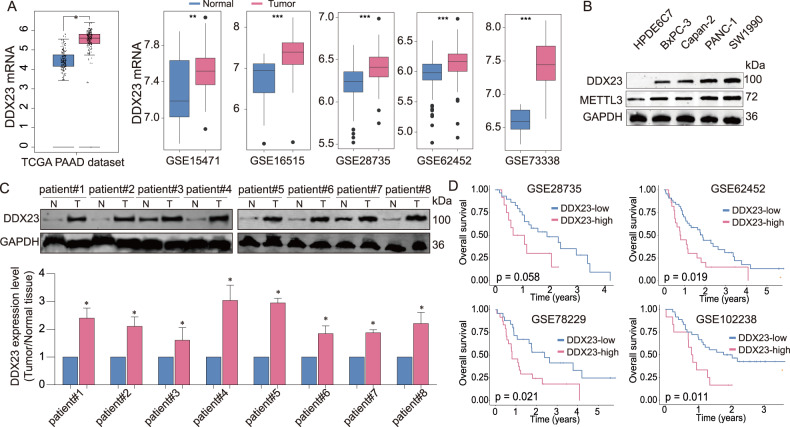
DDX23 was significantly upregulated in pancreatic cancer and predicted poor survival outcomes. **A** The differential gene expression analyses of TCGA and GEO pancreatic cancer data showed the significant upregulation of DDX23 in tumor tissues. **B** DDX23 protein expression in pancreatic cell lines was analyzed using western blotting assay. **C** DDX23 protein level in tumor and adjacent tissues of eight patients. **D** The survival analysis results of pancreatic cancer patients distinguished by DDX23 level. Datasets were obtained from the GEO database (https://www.ncbi.nlm.nih.gov/geo/).

To detect the correlation between DDX23 expression and PDAC progresson, we explored the expression of DDX23 in clinical tissue samples and the relationship with survival of PDAC patients. DDX23 mRNA was significantly upregulated in tumor tissues compared with the tumor-adjacent tissues (data obtained from the publicly available source, Fig. 4A). In line with this, we found the significant upregulation of DDX23 in pancreatic cancer cell lines compared with HPDE6C7 cell line (Fig. 4B). In addition, we validated the findings in our collected tissue samples (Fig. 4C; Fig. S4). Furthermore, in the four GEO datasets with survival data, we observed that patients with a higher level of DDX23 showed worse overall survival outcomes (Fig. 4D). Collectively, DDX23 was demonstrated to be significantly elevated in PDAC and potentially predicted a worse survival outcome.

### The knockdown of DDX23 led to suppressed pancreatic cancer cell progression and sensitivity to GEM

**Fig. 5:**
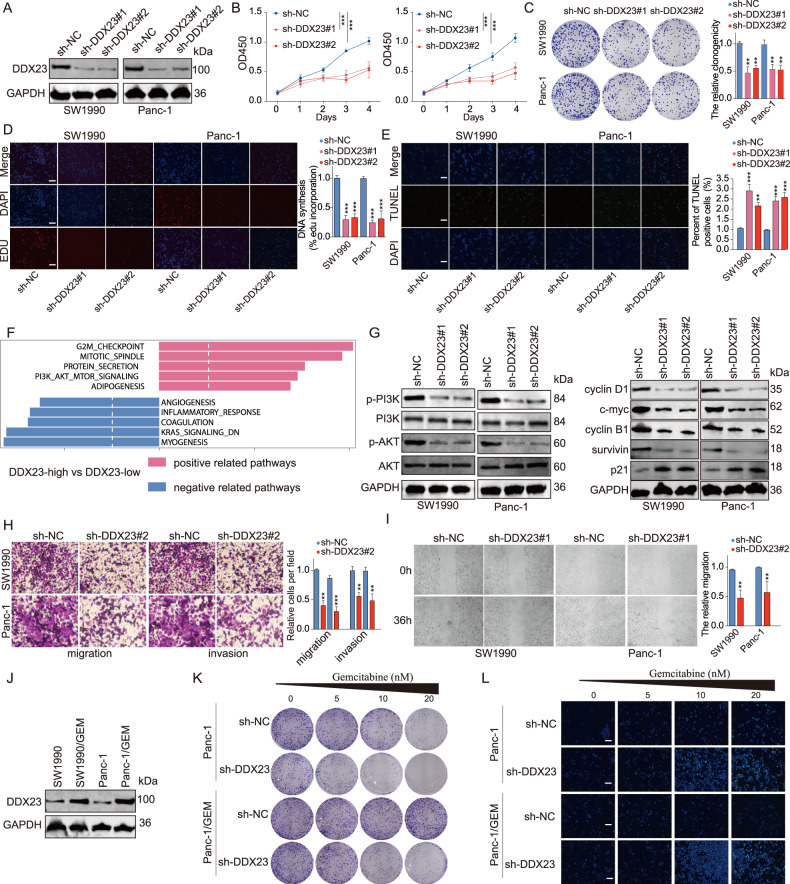
The knockdown of DDX23 significantly suppressed the proliferation, migration, invasion as well as resistance to gemcitabine of pancreas cancer cells via PI3K/Akt signaling. **A** The two shRNAs against DDX23 significantly suppressed the expression of DDX23 protein. **B** In the CCK8 assay, DDX23 knockdown inhibited the optical density (OD) at 450 nm in the two cell lines. **C**, **D** In colony formation assay (**C**) and EDU incorporation assay (**D**), DDX23 silence suppressed the proliferation of the two cell lines. **E** In the TUNEL assay, the knockdown of DDX23 led to a significantly higher TUNEL positive cell proportion. **F** The signaling pathway correlation analysis result showed that DDX23 expression was positively correlated with G2M checkpoint, mitotic spindle, protein secretion, PI3K/Akt signaling, and adipogenesis, and negatively correlated with angiogenesis, inflammatory response, coagulation, KRAS signaling, and myogenesis. **G** The knockdown of DDX23 led to significantly inhibited p-PI3K, p-Akt, cyclin D1, c-myc, cyclin B1, survivin levels but increased p21 expression in the two cell lines. **H** In the Transwell assay, DDX23 knockdown led to the suppression of migration and invasion of both cell lines. **I** In wound healing assay, DDX23 silence resulted in the suppression of cell migration in 36 h. **J** In GEM-resistant cell lines, DDX23 was significantly upregulated. **K** DDX23 knockdown led to suppressed cell growth in the Panc-1 cell line and GEM-resistant Panc-1 cell line. **L** In the TUNEL assay, DDX23 knockdown resulted in significantly enhanced TUNEL positivity in the Panc-1 cell line and GEM-resistant Panc-1 cell line. **P < 0.01, ***P < 0.001, compared with the sh-NC group.

Subsequently, we carried out a spectrum of experiments to study the role of DDX23 in PDAC. DDX23 was knockdowned by shRNA in PDAC cell lines (Fig. 5A). DDX23 silencing led to significantly suppressed cell proliferation (Fig. 5B–D), and more than two-fold cell apoptosis compared with the control group (Fig. 5E). We also discovered that DDX23 was positively associated with the G2M checkpoint pathway, mitotic spindle pathway, protein secretion pathway, PI3K/Akt signaling pathway, and adipogenesis according to the bioinformatics analysis results (Fig. 5F, Fig. S5). To further explore the effects of DDX23 on PI3K/Akt signaling, DDX23 loss-of-function experiments were conducted. DDX23 depletion led to significantly suppressed p-PI3K, p-Akt, cyclin D1, c-myc, cyclin B1, and survivin protein expression in the two cell lines (Fig. 5G). Meanwhile, Transwell and wound healing assay results revealed that DDX23 knockdown led to suppressed migration and invasion of the two cell lines by approximately 50% (Fig. 5H, I). Furthermore, the protein level of DDX23 was significantly higher in GEM-resistant cell lines compared with the parent cell lines (Fig. 5J). Subsequently, we carried out colony formation and TUNEL assays to learn the effects of DDX23 knockdown in GEM-resistant cell lines. Not surprisingly, in both the parent and GEM-resistant Panc-1 cell lines, DDX23 knockdown resulted in significantly suppressed colony formation (Fig. 5K) and increased apoptosis (Fig. 5L) in synergy with GEM treatment of different concentrations.

**Fig. 6:**
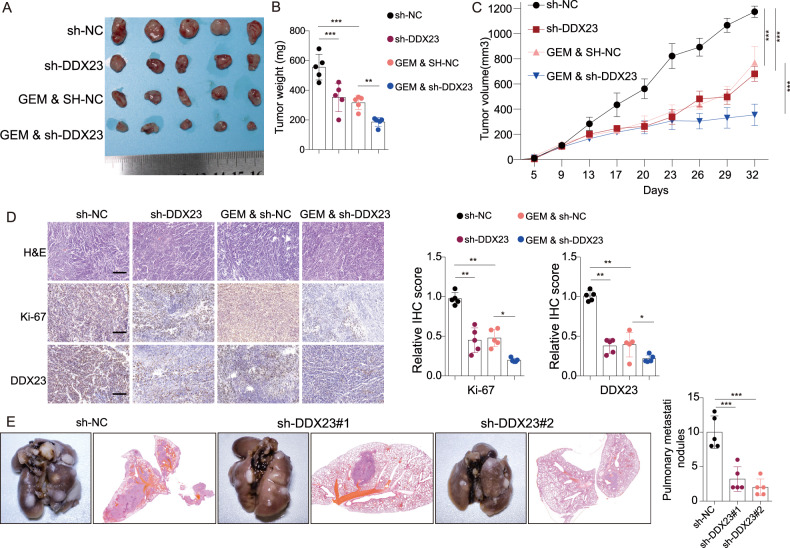
DDX23 knockdown led to suppressed in vivo tumor growth and lung metastasis. **A**–**C** The size (**A**), weight (**B**), and volume (**C**) of the in vivo tumors showed that DDX23 knockdown led to suppressed tumor growth in synergy with gemcitabine treatment. **D** H&E and Ki-67 IHC results showed that DDX23 knockdown led to suppressed tumor growth. **E** DDX23 knockdown led to suppressed lung metastasis, shown by the inhibited numbers of lung nodules. *P < 0.05, **P < 0.01, ***P < 0.001.

To further validate the potential tumor promotive role of DDX23, in vivo xenograft and lung metastasis experiments were conducted. DDX23 knockdown led to significantly smaller tumor size (Fig. 6A), lighter tumor weight (Fig. 6B), and smaller tumor volume (Fig. 6C) in synergy with GEM treatment. The H&E pathology and IHC analysis of tumor tissues also demonstrated that DDX23 silence resulted in less intense Ki-67 staining (Fig. 6D). In addition, xenograft models in the DDX23 knockdown group showed a significantly reduced (by more than 50%) number of pulmonary nodules (Fig. 6E), suggesting significantly suppressed lung metastasis. Besides of that, DDX23 depletion resulted in significantly suppressed phosphorylated PI3K and Akt expression in xenograft tumor tissues (Fig. S6). In summary, DDX23 knockdown could significantly suppress the malignant phenotypes of pancreatic cancer cells, in vivo tumor growth as well as lung metastasis in synergy with GEM treatment potentially by regulating PI3K/Akt signaling.

### DDX23 was responsible for the METTL3-induced aggressive tumor phenotypes

**Fig. 7:**
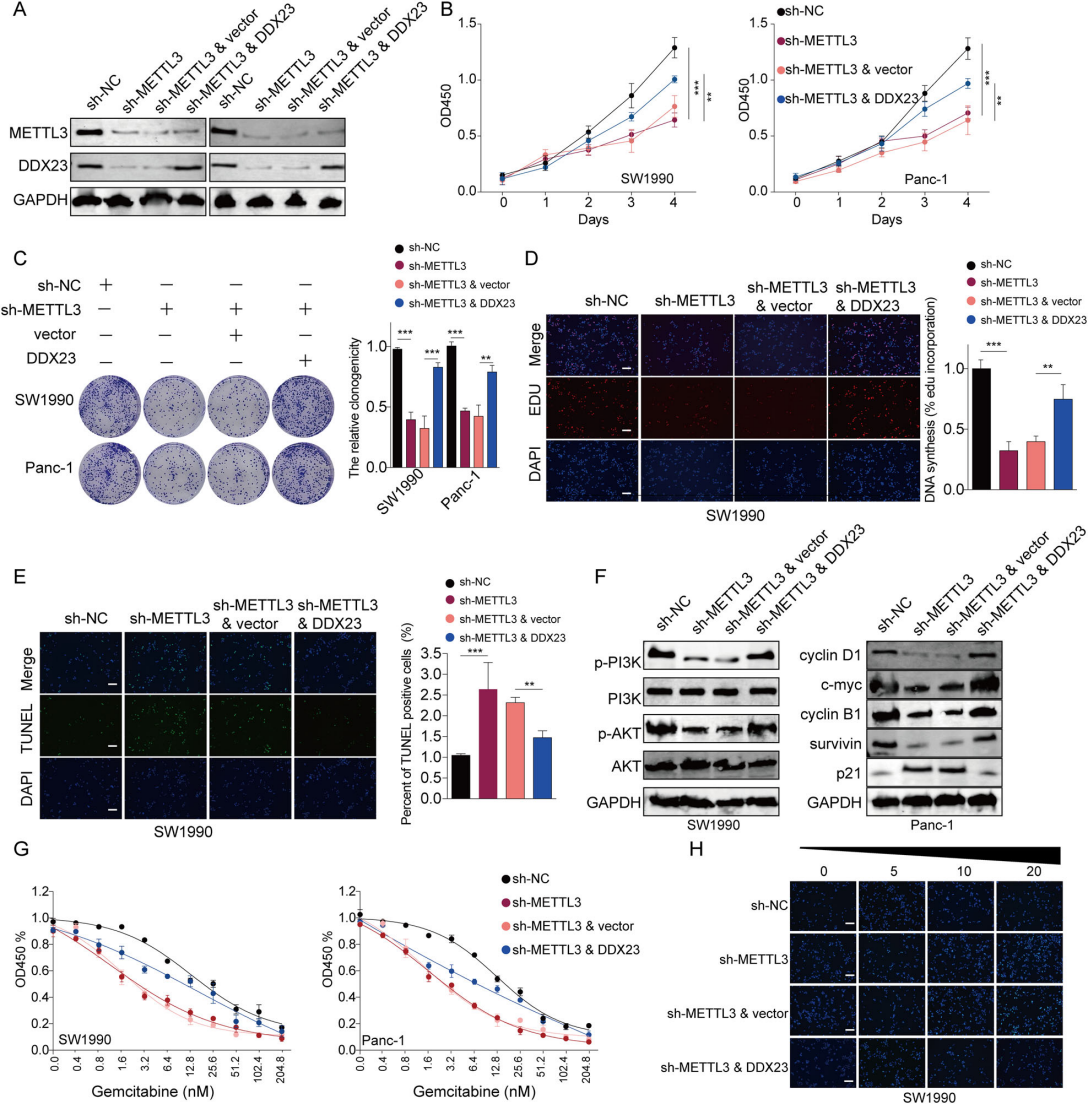
The effects of METTL3 knockdown were restored by the simultaneous upregulation of DDX23. **A** The knockdown of METTL3 and overexpression of DDX23 were confirmed at the translational level. **B** The suppressed OD values of the two cell lines by METTL3 knockdown were restored by the simultaneous overexpression of DDX23. **C**, **D** The suppressed cell proliferation resulting from METTL3 silence was partially restored by DDX23 upregulation. **E** The elevated cell apoptosis resulting from METTL3 knockdown was partially reversed by DDX23 demonstrated by the TUNEL assay. **F** The knockdown of METTL3 led to suppressed PI3K and Akt phosphorylation as well as cell cycle-related proteins, which were elevated by DDX23 overexpression. **G**, **H** DDX23 overexpression enhanced GEM resistance of cells with METTL3 knockdown, demonstrated by CCK8 assay (**G**) and TUNEL assay (**H**). **P < 0.01, ***P < 0.001.

**Fig. 8:**
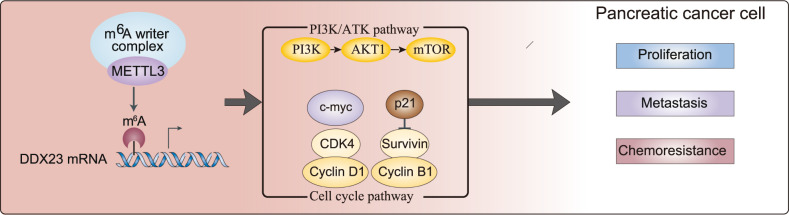
A scheme illustrating the potential mechanism of how METTL3 regulates pancreatic cancer progression by regulating PI3K/Akt signaling and cell cycle progression via m6A modifying DDX23 mRNA.

To further illuminate the role of DDX23 in METTL3-induced pancreatic cancer progression, we overexpressed DDX23 in PDAC cells depleted of METTL3. The downregulation of DDX23 protein by METTL3 knockdown was reversed by DDX23 overexpression (Fig. 7A). We found that DDX23 overexpression notably abrogated the inhibitive effects of METTL3 knockdown on the proliferation (CCK8 colometric assay, clone formation, and EDU incorporation rate) of SW1990 and Panc-1 cells (Fig. 7B–D). In addition, the enhanced cell apoptosis by METTL3 silence was suppressed by simultaneous overexpression of DDX23 by approximately 30% (Fig. 7E). Furthermore, the suppressed expression of p-PI3K, p-Akt, cyclin D1, c-myc, cyclin B1, and survivin by METTL3 silence was a significantly counteracted by DDX23 upregulation (Fig. 7F). We subsequently studied how DDX23 affected GEM resistance of pancreatic cancer in a METTL3-dependent manner. The CCK-8 assay results showed that the enforced DDX23 upregulation increased the IC_50_ of the two cell lines with METTL3 depletion (Fig. 7G). Also, the TUNEL assay results demonstrated that the knockdown of METTL3 enhanced the TUNEL fluorescence in the SW1990 cell line, suggesting the enhanced sensitivity to GEM of the cells (Fig. 7H). Besides of that, it was shown that METTL3 depletion resulted in suppressed Transwell migration and invasion (Fig. S7). These data suggested the function of METTL3 in promoting PDAC aggressive tumor phenotypes was partially depend on DDX23. To summarize, METTL3 modified DDX23 mRNA in an m6A modification manner thus promoting cancer cell proliferation, mobility, and chemoresistance through PI3K/Akt signaling (Fig. 8).

## Discussion

The m6A methyltransferase METTL3 was found to be upregulated in GEM-resistant pancreatic cancer cell lines, and its silence led to suppressed cancer cell proliferation and resistance to GEM. An identified downstream target of METTL3, DDX23 mRNA, was discovered to be upregulated in pancreatic cancer and predict poor survival, as well as positively correlated with PI3K/Akt pathway. In addition, the knockdown of DDX23 resulted in inhibited cancer cell proliferation and mobility as well as resistance to GEM. Lastly, the restoration experiments demonstrated that the inhibitive effects of METTL3 knockdown on cell phenotypes were compromised by the simultaneous upregulation of DDX23 potentially through PI3K/Akt signaling.

Aberrant m6A modification caused by the dysregulation of methyltransferases, demethylases, m6A-binding proteins, etc., is involved in the carcinogenesis of various cancers, including pancreatic cancer [13]. In addition, m6A modifications were reported to actively participate in the tumor microenvironment of pancreatic adenocarcinoma [14–17]. Thus, targeting methyltransferases such as METTL3 or their target transcripts seems to be a promising approach for pancreatic cancer therapeutics [18, 19], and there have been evidence supporting it. For instance, METTL3-mediated m6A modification on the 5’UTR of NUCB1 (encoding a calcium-binding protein) mRNA via YTHDF2 (YTH N6-methyladenosine RNA binding protein 2) reader protein in pancreatic adenocarcinoma was associated with poor prognosis. The upregulation of NUCB1 worked in synergy with gemcitabine and impaired pancreatic cancer cell proliferation [20]. Interestingly, METTL5 (a methyltransferase that specifically catalyzes 18 S rRNA N6 methylation at adenosine 1832) was considered an oncogene that promoted pancreatic cancer progression synergistically with its cofactor TRMT112 (tRNA methyltransferase activator subunit 11‑2) [21]. In addition, inhibited exon skipping of METTL14 and enhanced exon skipping of Cyclin L2 caused by CLK1’s enhancing SRSF5 SRSF5250-Ser phosphorylation enhanced the N6-methyladenosine modification level and cancer aggressiveness in pancreatic cancer [22]. Thus, the manipulation of methyltransferases expression significantly affected pancreatic cancer malignancy. This study found the significantly upregulated METTL3 in pancreatic cancer, and METTL3 direct target DDX23 mRNA and promotes its translation in a YTHDF1-dependent pathway. The forced silence of METTL3 and DDX23 led to suppressed cancer phenotypes and in vivo malignancy, showing the potential of METTL3/DDX23 axis as therapeutic targets for PDAC.

The role of DDX23, on the other hand, has not been elucidated clearly in cancers. No role description in pancreatic cancer has been reported. Yet, it was found to promote ovarian cancer progression by regulating the mRNA processing of FOXM1 [10]. DDX23, as a substrate protein, was reported to interact with SDC4 with the aid of bufalin, a small molecule anticancer drug, to induce inactivated matrix metalloproteinases and elevated p38/JNK MAPKs phosphorylation in hepatocellular carcinoma [23]. In addition, DDX23 was suspected to be involved in myelodysplastic syndrome. The pharmacological restoration of DDX23 might be a potential therapeutic approach for myelodysplastic syndrome [24]. Therefore, DDX23 is differentially expressed and demonstrates different roles in various human cancers. We herein, for the first time identified DDX23 to be a direct target of METTL3 and an oncogene in pancreatic cancer. The knockdown of DDX23 led to suppressed cancer malignancy, and tumor growth and metastasis. Besides, we found a close correlation between DDX23 upregulation and PI3K/Akt signaling, which has long been considered to be an oncogenic signaling pathway. Our study thus presented DDX23 to be a charming therapeutic target for pancreatic cancer drug development.

The correlation between m6A modification and PI3K/Akt signaling not only is involved in non-cancer disorders such as acute liver injury [25] and hypothermia [26] but also m6A RNA modifications were found to influence PI3K/Akt signaling in most human cancers, indicating a critical role in oncogenesis and a significant prognosis biomarker [27]. The suppression of m6A has shown promising effects in reducing oncogenesis. For instance, METTL14 was found downregulated and the m6A modification level was low in gastric cancer. The upregulation of METTL14 suppressed the malignant phenotypes by deactivating the PI3K/Akt/mTOR signaling [28]. Consistently, METTL14 knockdown promoted gastric cancer cell growth, whereas FTO knockdown (m6A upregulation) reversed the malignant phenotypes [29]. Also, downregulated METTL14 was associated with malignant phenotypes of hepatocellular cancer. Meanwhile, METTL14 affected HCC malignancy by regulating its direct target EGFR and PI3K/AKT signaling in an m6A-dependent manner [30]. Specifically, METTL3 was found upregulated in retinoblastoma, and its silence decreased retinoblastoma malignancy by regulating PI3K/Akt signaling, probably by affecting two downstream effectors P70S6K and 4EBP1 [31]. Thus, m6A modification by methyltransferases such as METTL14 and METTL3 may regulate oncogenesis by regulating PI3K/Akt signaling. Based on the findings in our study, we concluded that METTL3 regulated pancreatic cancer phenotypes by modifying its direct target DDX23 mRNA, thus PI3K/Akt signaling.

Gemcitabine resistance of pancreatic cancer is a prevalent symptom and needs to be addressed. It was reported that METTL14 was up-regulated in gemcitabine-resistant pancreatic cancer cells. METTL14 knockdown in vitro and in vivo impaired the gemcitabine resistance probably by suppressing cytidine deaminase expression [32]. Also, there has been evidence showing that microbiome treatment might increase the infiltration of GEM into the pancreatic tissue, thus improving the efficacy of GEM treatment. In this study, we found that the inhibition of METTL3 increased the sensitivity of pancreatic cancer cells to GEM. Yet, how METTL3 silence led to the improved efficacy of GEM treatment remains further investigated.

## Conclusion

To sum up, to address the severe gemcitabine resistance of pancreatic cancer, efforts have been made, especially in the discovery of novel and efficient therapeutic targets dependent on the m6A modification mechanism. Herein our team found a potential oncogenic circuit that involved PI3K/Akt signaling, a notorious oncogenic pathway. The knockdown of METTL3, an m6A methyltransferase, suppressed pancreatic cancer malignancy and gemcitabine resistance by regulating its direct target DDX23 mRNA, thus PI3K/Akt signaling. Our study may provide a new approach to addressing gemcitabine resistance as well as a new potential target axis for pancreatic cancer treatment.

## Methods

### Cells and reagents

Parent cell lines SW1990, Panc-1, and BxPC-3 were purchased from Procell (Wuhan, China). The HPDE6-C7 cell line and Capan-2 were maintained in our lab. GEM-resistant SW1990 and Panc-1 cell lines were established in our laboratory. Briefly, SW1990 and Panc-1 cell lines were exposed to a series of concentrations of gemcitabine (purity >98%; 5, 10, 20, 40, 80, 160, and 320 μM, Sigma-Aldrich) biweekly until cells became resistant to 320 μM of gemcitabine. BxPC-3 cell line was cultured in RPMI-1640 medium containing 10% FBS. SW1990, Panc-1, and the respective GEM-resistant cell lines were cultured in the DMEM medium containing 10% FBS, 100 U/mL of penicillin, and 100 mg/mL of streptomycin at 5% CO_2_, 37 °C.

### Cell transfection

The shRNA constructs were generated by inserting shRNA fragments (of METTL3, DDX23) into the shLenti vectors (constructs were purchased from Origene, Rockville, MD, USA), all shRNA sequence used are provided in Supplementary Table S1. The overexpression constructs were generated by inserting the fragments of the full-length coding region of DDX23 into pcDNA3.1 plasmids. The functional constructs, nontarget shRNA (Sigma-Aldrich), and negative control of the corresponding constructs were diluted in the medium containing 6 μg/mL polybrene. The transfection of constructs into cells was performed using Lipofectamine 2000 (Invitrogen). Three days after the transfection, 5 μg/mL of puromycin was added to select successfully transfected cells.

### mRNA stability analysis and RT-qPCR

Total RNA was extracted from the cells using TRIzol (Beyotime, Shanghai, China) and treated with DNase I (Sigma-Aldrich). M6A colorimetric quantification was performed to analyze the cellular m6A level using commercialized kit (EpiGentek, USA). The process was carefully handled following the manual of the kit. In addition, mRNA stability was analyzed based on a previously published protocol [33]. Briefly, the transfected cells were harvested or treated with 5 mM Actinomycin D and harvested 6 and 12 h after the treatment. RNA was reverse transcribed into cDNA; thereafter, the fluorescence PCR reaction was carried out using SYBR Green I reagent (Solarbio Life Sciences, Beijing, China) in a Real-Time PCR System (Applied Biosystems, USA). The primer sequences used in this study were shown in Supplementary Table S2.

### Immunoblotting assay

Standard western blotting assays were conducted. GAPDH was used as the loading control. Briefly, the cells were harvested and lysed in RIPA buffer with protease inhibitor on ice. Protein concentrations were determined using the bicinchoninic acid method. Subsequently, the proteins were separated by 10% SDS-PAGE and transferred onto a nitrocellulose membrane (Sigma-Aldrich). The proteins of interest were hybridized with the corresponding primary and secondary antibodies. Then, the chemiluminescence reagents were added before the protein blotting bands were visualized. The relative levels of METTL3, DDX23, and PI3K/Akt signaling-related proteins such as p-PI3K, PI3K, p-Akt, and Akt as well as cell cycle-related proteins such as cyclin D1, c-myc, cyclin B1, survivin and p21 were reflected by reading the intensity of the immunoblotting bands using Image J software. All antibodies used are provided in Supplementary Table S3. All uncropped western blots are provided in Fig. S8.

### Proliferation assay

Approximately 4000 cells/well were seeded into a 96-well plate and incubated with different concentrations of gemcitabine for 1 day. The optical absorbance at 450 nm of cells was detected after the addition of 10 μL CCK8 reagent for 1 h. The IC50 value was thus obtained from the curve. All assays were performed in triplicate. Colony formation assays were also conducted to determine cell proliferation under different treatment conditions. Approximately 2000 cells were plated into six-well plates and cultured for 1 day. Thereafter, the cells were treated with gemcitabine or shRNA constructs for 24 h. The fresh medium was added to the cells and further incubated for 2 weeks before the cells were fixed with 4% formaldehyde and stained with 0.5% crystal violet. The number of colonies was then counted under a microscope. EDU incorporation assays were carried out to determine cell proliferation phenotype. Briefly, the cells were cultured in a 24-well plate for 1 day. EDU assay kit (Ribobio) containing DAPI that was used to stain the nuclei was used to perform the incorporation assays. An Olympus fluorescent microscope was used to observe the proliferating cells.

### TUNEL assay

The cell suspension was prepared and fixed in 4% paraformaldehyde for 0.5 h and washed in PBS with 0.3% Triton X-100. The TUNEL mixture, including the TdT enzyme and fluorescence labeling solution was prepared according to the manual of the TUNEL assay kit (C1086, Beyotime). Then, 50 μl of the TUNEL mixture was mixed with the cell suspension and incubated at 37 °C for 1 h. Lastly, the cell nuclei were dyed with DAPI, and cells with TUNEL labeling were observed under the fluorescent microscope at 100×. Three random fields were selected from each group.

### Transwell assay

24-well Transwell inserts with 8-μm pore size (Corning Inc., USA) were used to determine cell migration and invasiveness. The inserts were pre-coated with extracellular matrix gel for invasiveness but not for the migration experiments. The cells were cultured in the serum-free medium overnight and resuspended in the medium containing FBS. The cells were then seeded into the Transwell inserts in triplicate to be allowed to migrate into the lower chambers that were filled with the DMEM medium containing 10% FBS (as the attractant). 36 h later, the cells on the lower surface were fixed and stained. Five random microscopic fields were selected and photographed. The stained cells in every field were counted. The wound-healing assay was employed to determine the migration capability of cells. Briefly, cells with different treatments were cultured to form monolayers in 6-well plates until 90–100% confluence. Thereafter, the cell layers were scratched with sterile plastic tips and washed with PBS to get rid of the loose cells. The cells were then cultured for a further 36 h and finally photographed using a microscope.

### Xenograft and lung metastasis assay

Six***-***week-old female BALB/c nude mice were purchased from Beijing Vital River Laboratory Animal Technology Co., Ltd (Beijing, China). SW1990 cells (1 × 10^6^ cells/200 μl) transfected with shRNAs against METTL3 (sh-METTL3) or DDX23 (sh-DDX23) and the corresponding negative controls of shRNAs (sh-NC) were injected into the right flanks of the mice models. When the tumors grew into approximately 100 mm^3^ in volume, the mice were treated with 50 mg/kg gemcitabine every 4 days for 32 days. The tumor volumes were recorded every 4 days. Tumor volume = ab^2^/2 (a, the largest diameter of the tumor; b, the smallest diameter of the tumor). The tumors were resected from the mice models, fixed in formalin, and embedded with paraffin for H&E as well as immunohistochemistry (IHC) analyses. H&E staining was done to the tumor tissues and lung tissues of the mice models. IHC staining of METTL3, DDX23, and Ki67 was performed using the IHC reagents (Beyotime). The process was performed according to the manufacturer’s instructions. Sections were semi-quantitatively using the ImageJ plugin IHC profiler (NIH) version 10.2. The Ethics Committee of The first affiliated hospital of Fujian Medical University approved our animal-related protocols.

### Luciferase reporter assay

The fragments of DDX23 mRNA that contained the methylation sites of METTL3 were cloned into a pGL3 plasmid. The mutated sequence was synthesized by GenePharma (Shanghai, China). Luciferase activity was determined using the dual-luciferase reporter assay system (Promega, USA). The relative luciferase activity, represented by the ratio of firefly luciferase activity and Renilla luciferase activity was determined using a GloMax 20/20 Luminometer (Promega).

### Fluorescence in situ hybridization

The intensity of METTL3 and DDX23 in cells treated with METTL3 knockdown was determined using the fluorescence in situ hybridization assay. The cells were fixed in 4% paraformaldehyde shortly and rinsed with cold PBS before they were treated with 0.5% TritonX-100 (Beyotime) for 20 min. Subsequently, the cells were incubated in the hybridization buffer overnight. The next day, Cy3-labeled METTL3 probes and FITC-labeled DDX23 probes (Geneseed) were used to hybridize. DAPI (4,6-diamidino-2-phenylindole, C1006, Beyotime) was used to stain cell nuclei. The cells were observed and photographed after they were briefly rinsed using PBS.

### Bioinformatical and statistical analysis

Bioinformatical analyses were used in finding the potential downstream target of METTL3, studying the correlation between METTL3 expression and DDX23 expression, the relationship between DDX23 level and the overall survival of patients, the significantly DDX23-correlated signaling pathways. Data used in bioinformatical analyses were obtained from public databases, including m6a2target, TCGA, and GEO. For the experimental data, three independent experiments were conducted, and the statistical analyses were done using the Student’s t-test or one-way ANOVA dependent on the number of groups. *P* < 0.05 was considered to be significant.

## Supplementary information

Supplementary Figure Legends

Supplementary Table S3

Supplementary Figure S1

Supplementary Figure S2

Supplementary Figure S3

Supplementary Figure S4

Supplementary Figure S5

Supplementary Figure S6

Supplementary Figure S7

Supplementary Figure S8

Supplementary Table S1

Supplementary Table S2

Check list

aj-checklist

BXPC-3-STR

panc-1-STR

sw1990-STR

## Data Availability

The datasets used and/or analyzed during the current study are available from the corresponding author on reasonable request.
