# Correction: METTL3 enhances pancreatic ductal adenocarcinoma progression and gemcitabine resistance through modifying *DDX23* mRNA N6 adenosine methylation

**DOI:** 10.1038/s41419-023-05939-1

**Published:** 2023-07-31

**Authors:** Chengjie Lin, Ting Li, Yan Wang, Shihui Lai, Yue Huang, Zhenyun Guo, Xiang Zhang, Shangeng Weng

**Affiliations:** 1 Department of Hepatopancreatobiliary Surgery, The First Affiliated Hospital of Fujian Medical University, Fuzhou, Fujian 350001 China; 2 Fujian Abdominal Surgery Research Institute, The First Affiliated Hospital, Fujian Medical University, Fuzhou, Fujian 350001 China; 3 National Regional Medical Center, Binhai Campus of the First Affiliated Hospital, Fujian Medical University, Fuzhou, Fujian 350212 China; 4 Department of Oncology, Fujian Provincial Hospital, Provincial Clinical College of Fujian Medical University, Fuzhou, Fujian 350001 China

**Keywords:** Cancer genetics, Cell death

Correction to: *Cell Death and Disease* 10.1038/s41419-023-05715-1, published online 28 March 2023

In this article the funding note has been given erroneously:

This study was supported by grants: (I) Youth Scientific Reseach Project of Fujian Province Health Commission (2020QNA05*2*);

But should be read:

This study was supported by grants: (I) Youth Scientific Reseach Project of Fujian Province Health Commission (2020QNA05*1*);

The original article has been corrected.

