# Correction: METTL3 enhances pancreatic ductal adenocarcinoma progression and gemcitabine resistance through modifying *DDX23* mRNA N6 adenosine methylation

**DOI:** 10.1038/s41419-025-08135-5

**Published:** 2026-02-25

**Authors:** Chengjie Lin, Ting Li, Yan Wang, Shihui Lai, Yue Huang, Zhenyun Guo, Xiang Zhang, Shangeng Weng

**Affiliations:** 1 Department of Hepatopancreatobiliary Surgery, The First Affiliated Hospital of Fujian Medical University, Fuzhou, Fujian 350001 China; 2 Fujian Abdominal Surgery Research Institute, The First Affiliated Hospital, Fujian Medical University, Fuzhou, Fujian 350001 China; 3 National Regional Medical Center, Binhai Campus of the First Affiliated Hospital, Fujian Medical University, Fuzhou, Fujian 350212 China; 4 Department of Oncology, Fujian Provincial Hospital, Provincial Clinical College of Fujian Medical University, Fuzhou, Fujian 350001 China

**Keywords:** Cancer genetics, Cell death

Correction to: *Cell Death and Disease* 10.1038/s41419-023-05715-1, published online 28 March 2023

During the final figure assembly and layout process using Adobe Illustrator, an unfortunate technical error occurred. Specifically, the same data image was inadvertently duplicated and assigned to two different experimental groups in Fig. 7H. This was purely an error in the figure assembly and labeling process and does not reflect an error in the underlying experimental data or data analysis. The image currently representing group-sh-METTL3 & DDX23 in Fig. 7H is incorrect identical to the image representing group-sh-NC in Fig. 7H.

**Original Figure 7** 
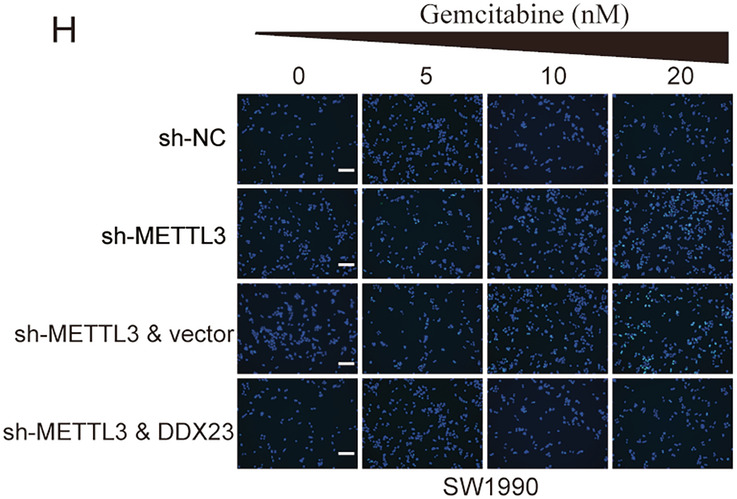


**Amended Figure 7** 
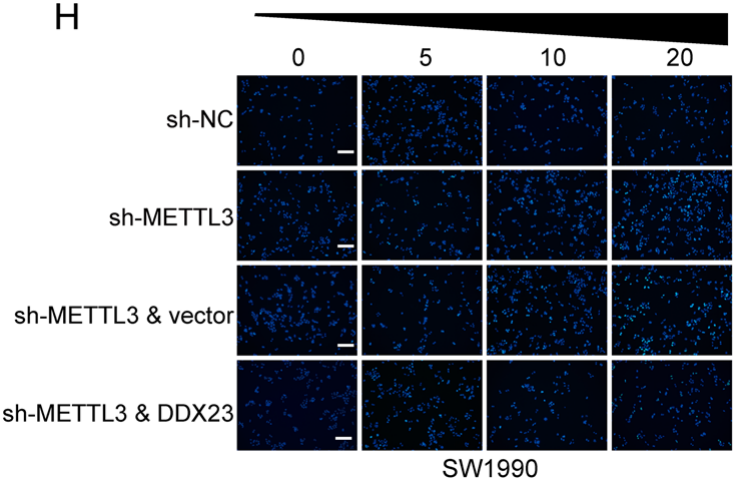


The original article has been updated.

