# Supplementary material for: METTL3 enhances pancreatic ductal adenocarcinoma progression and gemcitabine resistance through modifying DDX23 mRNA N6 adenosine methylation
Source: Cell Death Dis. 2023 Mar 28;14(3):221. doi: 10.1038/s41419-023-05715-1 (PMC10050319; doi:10.1038/s41419-023-05715-1)
Supplement: Supplementary file 1 — Supplementary Figure Legends [file 41419_2023_5715_MOESM1_ESM.docx]

Supplementary Figure legends

**Supplementary Figure S1.** (a) western blotting confirmed efficient METTL3 knockdown in normal pancreas ductal epithelial cells HPDE6-C7. (b) CCK-8 assay were used to determine the viability of HPDE6-C7 after METTL3 silencing.

**Supplementary Figure S2.** (a) Schematic presentation of proposed mutation site for disruption of catalytic site of METTL3 protein. (b) The expression of DDX23 in Panc-1 and SW1990 cells transfected with empty vector (vector), wide type-METTL3 (METTL3-wide) or catalytic mutant METTL3 (METTL3-Mutant) was analyzed by western blotting;

**Supplementary Figure S3.** (a) The expression of DDX23 in PDAC cells transfected with si-YTHDF2, si-YTHDF1 or si-NC was analyzed by western blotting; (b) The expression of DDX23 in BxPC-3 cells transfected with negative control (NC), METTL3 plasmid (METTL3) with si-NC or si-YTHDF1, was analyzed by western blotting.

**Supplementary Figure S4.** Representative figures for DDX23 in PDAC tissues and adjacent tissues using IHC staining. Scale bar: 200 μm.

**Supplementary Figure S5. Signaling pathway enrichment analysis.** A. The signaling pathway correlation analysis shows that DDX23 activation is positively correlated with mitotic spindle, E2F targets, G2M checkpoint, and PI3K/Akt signaling *etc.*. B. GSEA analysis of the correlation between PI3K/Akt/mTOR signaling and DDX23 expression.

**Supplementary Figure S6.** IHC results of phosphorylated PI3K and Akt expression in xenograft tumor tissues. n=5. ^**^*P*<0.01, compared with sh-NC group.

**Supplementary Figure S7.** The suppressive effect of METTL3 knockdown was counteracted by the simultaneous upregulation of DDX23 in Transwell migration and invasion assays. ^**^*P*<0.01, ^***^*P*<0.001, compared with sh-NC group.
