# Supplementary material for: METTL3 enhances pancreatic ductal adenocarcinoma progression and gemcitabine resistance through modifying DDX23 mRNA N6 adenosine methylation
Source: Cell Death Dis. 2023 Mar 28;14(3):221. doi: 10.1038/s41419-023-05715-1 (PMC10050319; doi:10.1038/s41419-023-05715-1)
Supplement: Supplementary file 2 — Supplementary Table S3 [file 41419_2023_5715_MOESM2_ESM.docx]

**Supplementary Table S3. Antibodies used in this study**

| **Antibody** | **Manufacturer** | **Cat. No** |
| --- | --- | --- |
| GAPDH | HUABIO | ER1706-83 |
| METTL3 | Proteintech | 15073-1-AP |
| DDX23 | Abcam | ab70459 |
| p-PI3K | Abcam | ab278545 |
| p-AKT | Cell Signaling | 9271 |
| PI3K | Proteintech | 21739-1-AP |
| AKT | Proteintech | 10176-2-AP |
| Cyclin D1 | Abcam | ab16663 |
| C-MYC | Abcam | ab168727 |
| Cyclin B1 | Proteintech | 67686-1-Ig |
| Survivin | Santa Cruz | sc-17779 |
| P21 | Abcam | ab212247 |
