# Supplementary figures and images for: METTL3 enhances pancreatic ductal adenocarcinoma progression and gemcitabine resistance through modifying DDX23 mRNA N6 adenosine methylation
Source: Cell Death Dis. 2023 Mar 28;14(3):221. doi: 10.1038/s41419-023-05715-1 (PMC10050319; doi:10.1038/s41419-023-05715-1)

A

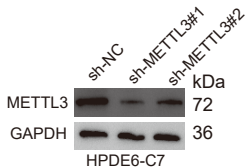

B

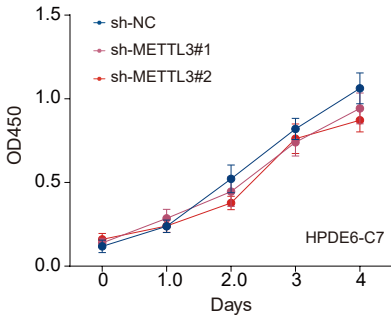

Supplement: Supplementary file 3 — Supplementary Figure S1 [file 41419_2023_5715_MOESM3_ESM.pdf]

A

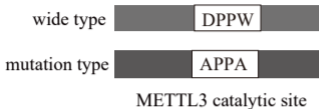

B

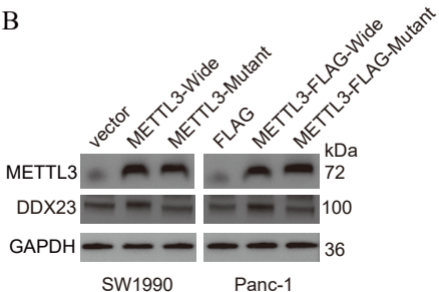

Supplement: Supplementary file 4 — Supplementary Figure S2 [file 41419_2023_5715_MOESM4_ESM.pdf]

A

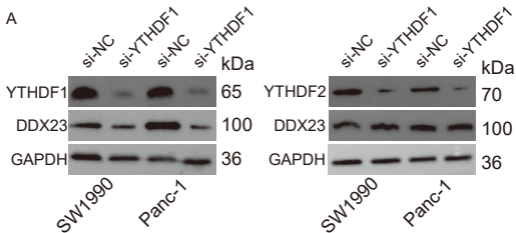

B

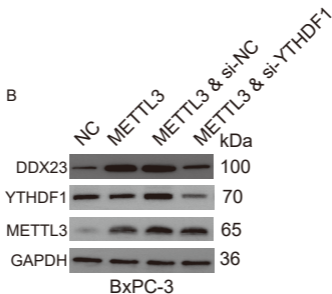

Supplement: Supplementary file 5 — Supplementary Figure S3 [file 41419_2023_5715_MOESM5_ESM.pdf]

adjacent tissues

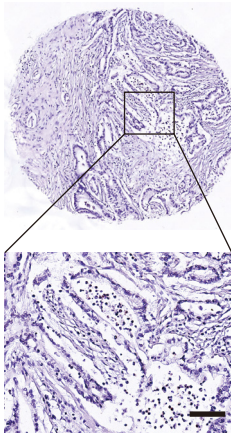

PDAC tissues case#1

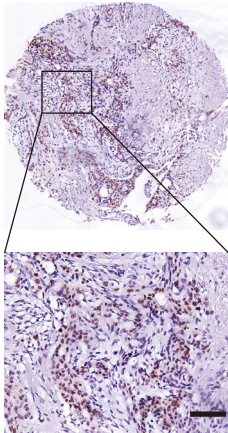

PDAC tissues case#2

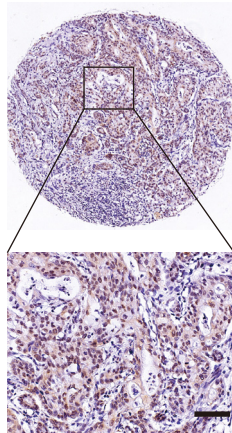

DDX23 IHC staining

Supplement: Supplementary file 6 — Supplementary Figure S4 [file 41419_2023_5715_MOESM6_ESM.pdf]

A

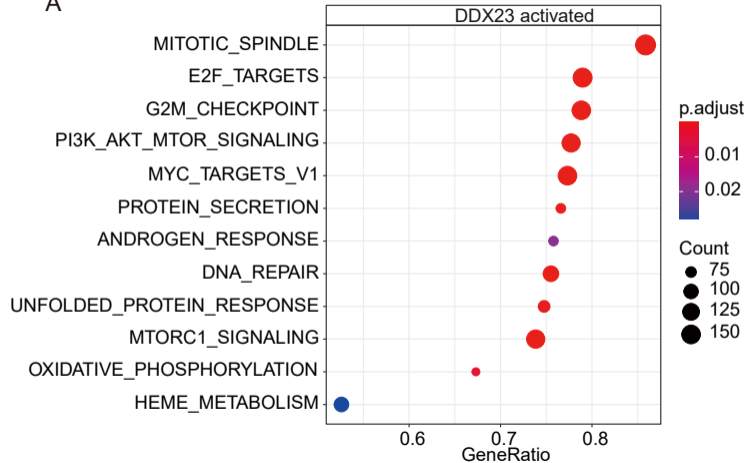

B

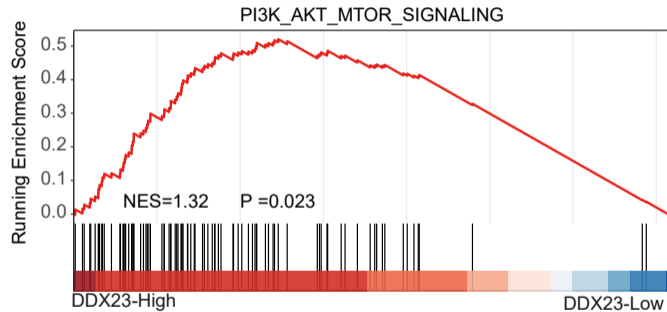

Supplement: Supplementary file 7 — Supplementary Figure S5 [file 41419_2023_5715_MOESM7_ESM.pdf]

p-PI3K

p-AKT

sh-NC

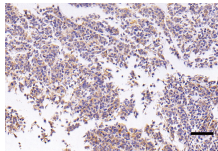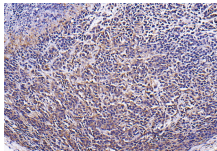

sh-DDX23

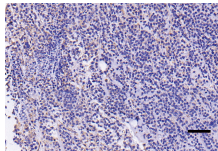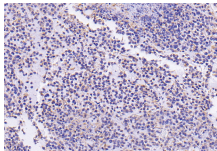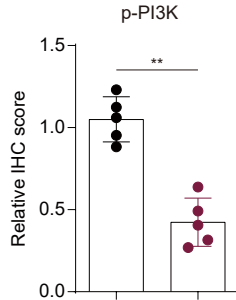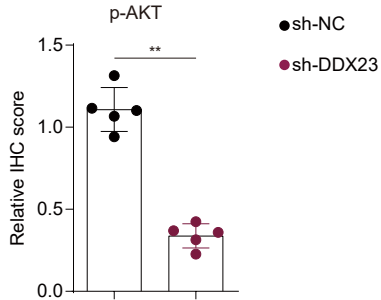

Supplement: Supplementary file 8 — Supplementary Figure S6 [file 41419_2023_5715_MOESM8_ESM.pdf]

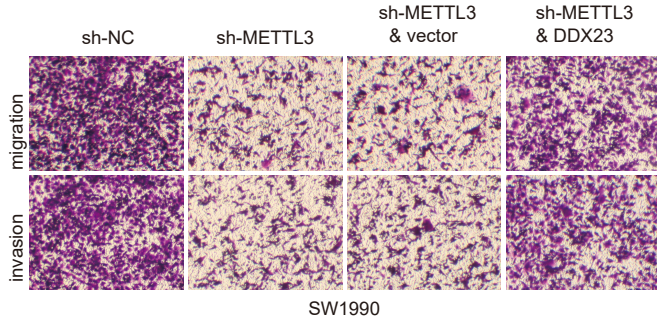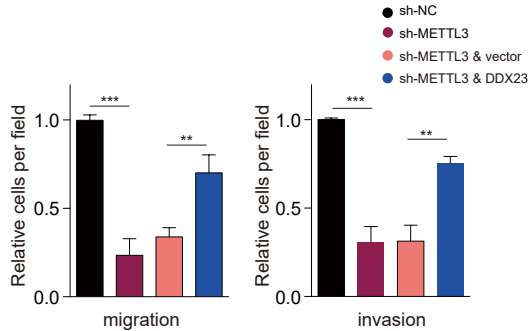

Supplement: Supplementary file 9 — Supplementary Figure S7 [file 41419_2023_5715_MOESM9_ESM.pdf]

Fig.1A

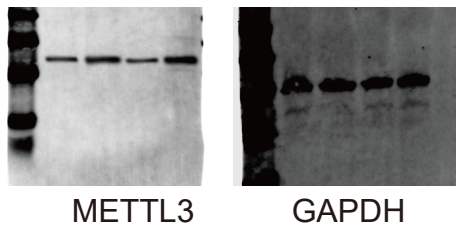

Fig.1E

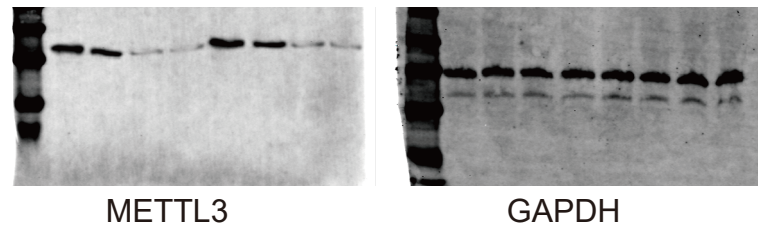

Fig.3E

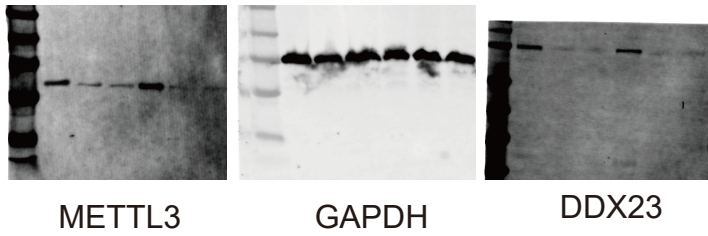

Fig.3F

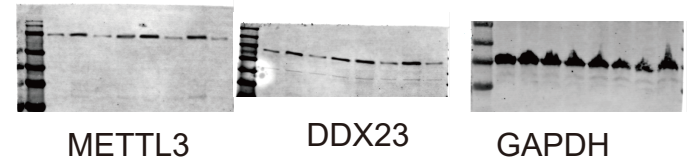

Fig.4C

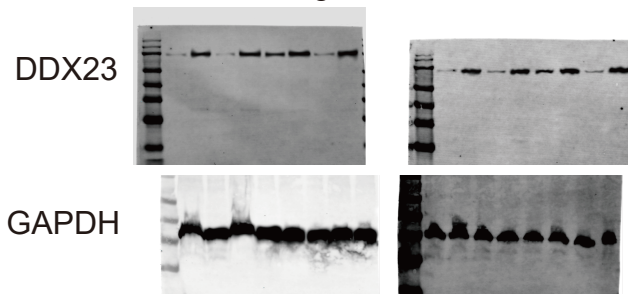

Fig.5A

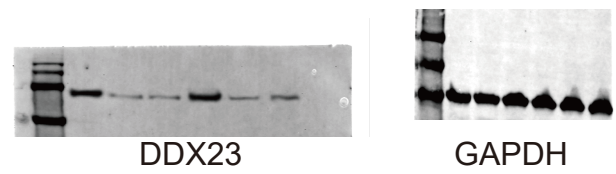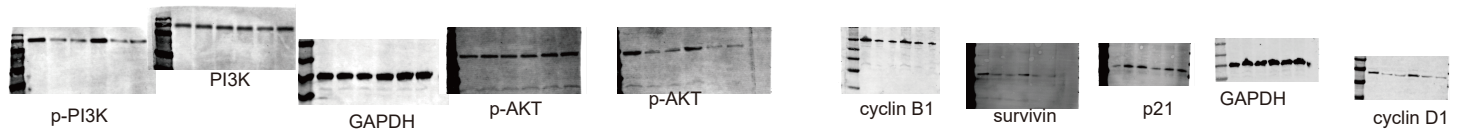

Fig.5G

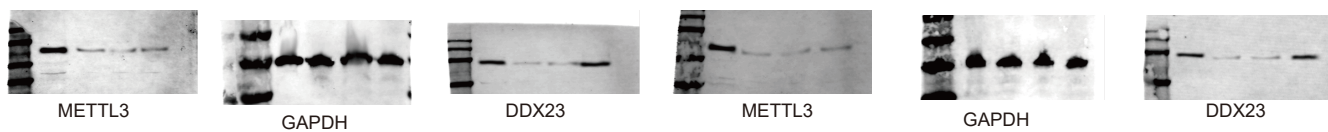

Fig.7A

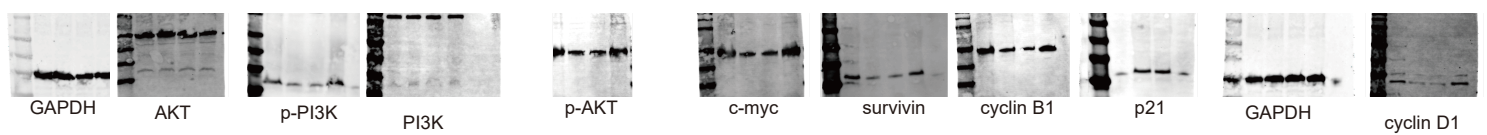

Fig.7F

Supplement: Supplementary file 10 — Supplementary Figure S8 [file 41419_2023_5715_MOESM10_ESM.pdf]
