# Supplementary material for: METTL3 enhances pancreatic ductal adenocarcinoma progression and gemcitabine resistance through modifying DDX23 mRNA N6 adenosine methylation
Source: Cell Death Dis. 2023 Mar 28;14(3):221. doi: 10.1038/s41419-023-05715-1 (PMC10050319; doi:10.1038/s41419-023-05715-1)
Supplement: Supplementary file 11 — Supplementary Table S1 [file 41419_2023_5715_MOESM11_ESM.docx]

**Supplementary Table S1. shRNA sequence**

| Name | Primer (5'-3') |
| --- | --- |
| Sh-METTL3#1 | CACCGGGCCCAAGTGCAAGAATTCTCGAAAGAATTCTTGCACTTGGGCCC |
| Sh-METTL3#2 | CACCGCCCAAGTGCAAGAATTCTGTCGAAACAGAATTCTTGCACTTGGGC |
| Sh-DDX23#1 | CACCGGAAGGACAGAGACTCTAAGACGAATCTTAGAGTCTCTGTCCTTCC |
| Sh-DDX23#2 | CACCGGATGAAGAGGATGAACATGGCGAACCATGTTCATCCTCTTCATCC |
| Sh-NC | CCGGCGTGATCTTCACCGACAAGATCTCGAGATCTTGTCGGTGAAGATCTTTTT |
