# Supplementary material for: METTL3 enhances pancreatic ductal adenocarcinoma progression and gemcitabine resistance through modifying DDX23 mRNA N6 adenosine methylation
Source: Cell Death Dis. 2023 Mar 28;14(3):221. doi: 10.1038/s41419-023-05715-1 (PMC10050319; doi:10.1038/s41419-023-05715-1)
Supplement: Supplementary file 12 — Supplementary Table S2 [file 41419_2023_5715_MOESM12_ESM.docx]

**Supplementary Table S2. Primer sequence**

| Name | Direction | Primer (5'-3') |
| --- | --- | --- |
| DDX23 | Forward | GCGATCACGGACTCCTGAC |
|  | Reverse | GAACGATGCCGCTTTCTATCT |
| GAPDH | Forward | ACAACTTTGGTATCGTGGAAGG |
|  | Reverse | GCCATCACGCCACAGTTTC |
| SETD1A | Forward | CAGTGGCGGAACTACAAGCTC |
|  | Reverse | CATAGCGGTACACCTTCTGAGA |
| ZNF777 | Forward | CAGAGCCCGGTTTCCCAAAA |
|  | Reverse | TCGCAGTCGGCTATCTTCTTT |
| CIC | Forward | GGTGGAATCTGGTAAGGGTCC |
|  | Reverse | AACTGGATCTCAGGAGACATGA |
| RMB14 | Forward | ACTTGGAAGATTTTCGTGGGC |
|  | Reverse | CGGAACCATACCCTTGGTGG |
