# Supplementary material for: METTL3 enhances pancreatic ductal adenocarcinoma progression and gemcitabine resistance through modifying DDX23 mRNA N6 adenosine methylation
Source: Cell Death Dis. 2023 Mar 28;14(3):221. doi: 10.1038/s41419-023-05715-1 (PMC10050319; doi:10.1038/s41419-023-05715-1)
Supplement: Supplementary file 13 — Check list [file 41419_2023_5715_MOESM13_ESM.docx]

**ORIGINAL ARTICLE SUBMISSION CHECKLIST**

1. Cover Letter
- Declaration not submitted elsewhere
- Concise description of major findings
- Suggest potential reviewers to include or exclude
2. Detailed Attribution of Authorship
- Contribution to preparation of manuscript
- Detailed preparation of figures
3. Title page (excluding acknowledgements)
4. Abstract
- 300 words maximum
5. Introduction
6. Materials (or Subjects) & Methods
7. Results
8. Discussion
9. Acknowledgements
- Include all funding sources
10. Conflict of Interest
- Err on the side of fulldisclosure
11. References
12. Figure Legend
- Where appropriate, declare N
- Define error bars
- Define scale bars
13. Tables
14. Figures
- 6-8 figures
- Where appropriate, include molecular weight markers
- Where appropriate, include scalebars
- Manipulate images as little aspossible
15. Supplementary Information
