# Supplementary material for: METTL3 enhances pancreatic ductal adenocarcinoma progression and gemcitabine resistance through modifying DDX23 mRNA N6 adenosine methylation
Source: Cell Death Dis. 2023 Mar 28;14(3):221. doi: 10.1038/s41419-023-05715-1 (PMC10050319; doi:10.1038/s41419-023-05715-1)

细胞遗传质量鉴定检测

Cell Line Authentication Service

STR 基因型检测报告

样 品 信 息

样品编号：

客户样本编号	公司编号
Bxpc-3	20170912-02

样品数量： 1

样品性状： 细胞系

检测项目： STR

送检单位： 富衡

检测方法：用 Axygen 的基因组抽提试剂盒提取 DNA，采用 20- STR 扩增方案扩增，在 ABI 3730XL 型遗传分析仪上对 STR 位点和性别基因 Amelogenin 进行检测。

检 测 结 果

(一) 检验基本情况

	多等位基因	匹配细胞系	细胞库	EV 值	匹配说明
20170912-02	有	Bxpc-3	ATCC	1.00	完全匹配

样本基因型检验结果

- 多等位基因指三等位及以上基因现象。
- 本次检测各细胞分型结果良好。

(二) 各样本描述

- 20170912-02：该株细胞 DNA 分型在细胞系检索中找到**完全匹配**的细胞系，ATCC 数据库显示细胞名为 **bxpc-3**，细胞号对应 **CRL-1687**。本次检测在该细胞系中**发现多等位基因**。

备注：待测细胞系与收录于 ATCC, DSMZ, JCRB 和 RIKEN 数据库的细胞系 STR 数据进行比对，未收录于以上细胞库的细胞系将无法匹配。

（三）样本分型结果

**细胞** 20170912-02 **的 STR 位点和 Amelogenin 位点的基因分型结果**						
Loci	送检细胞 STR 信息			细胞库细胞 STR 信息		
	送检细胞名：bxpc-3			细胞库细胞名：bapc-3		
	Allele1	Allele2	Allele3	Allele1	Allele2	Allele3
D5S818	11	11		11	11	
D13S317	11	11		11	11	

D7S820	10	13		10	13	
D16S539	9	11		9	11	
VWA	14	18		14	18	
TH01	9	9		9	9	
AMEL	X	X		X	X	
TPOX	8	8		8	8	
CSF1PO	13	13		13	13	
D12S391	20	20				
FGA	22	25				
D2S1338	17	19				
D21S11	29	29				
D18S51	12	12				
D8S1179	13	13				
D3S1358	14	16				
D6S1043	12	12				
PENTAE	12	14				
D19S433	13	16.2				
PENTAD	14	14				

其他说明

(一) 分型方案及位点分布

	方案 1	方案 2	方案 3	方案 4

1	D3S1358	D8S1179	D19S433	AMEL
2	VWA	D21S11	TH01	D5S818
3	D7S820	D16S539	D13S317	D12S391
4	CSF1PO	D2S1338	TPOX	FGA
5	PENTAE	PENTAD	D18S51	
6			D6S1043	

实验方案及位点

(二) STR 数据库比对

本公司采用 DSMZ tools 进行细胞系比对，其中包含来自于 ATCC, DSMZ, JCRB 和 RIKEN 数据库的 2455 个细胞系 STR 数据。如果待检测细胞未收录于以上细胞库或这是自行建立的新细胞系将无法进行比对，用户需根据细胞分型结果自行与其他数据库进行比对。

签发日期：2017 年 9 月

Applied Biosystems
GeneMapper 4.0

Cell Line Authentication 0920

Sample File	Sample Name	Panel	OS	SQ
11_C02.fsa	Bxpc-3	STR Profile 1-human		

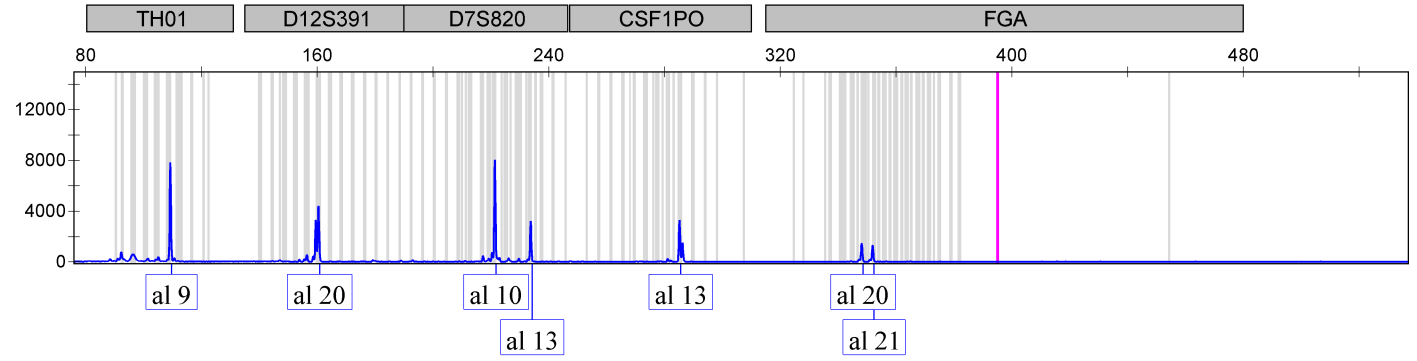

11_C02.fsa	Bxpc-3	STR Profile 1-human		

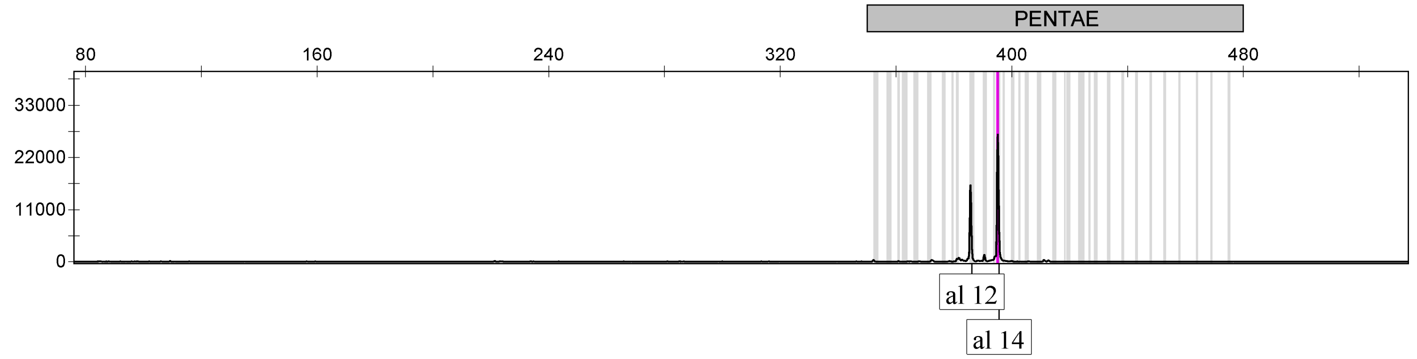

35_C05.fsa	Bxpc-3	STR Profile 2-human		

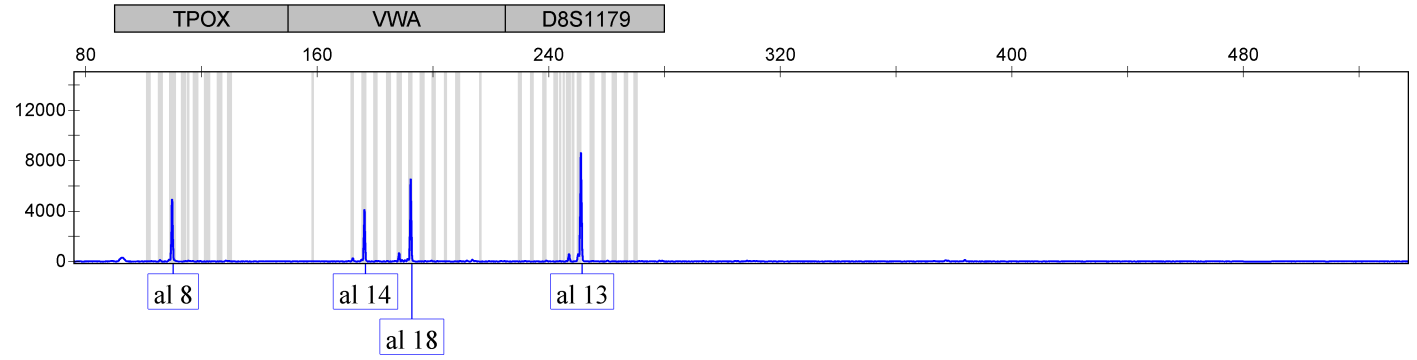

Sample File	Sample Name	Panel	OS	SQ
35_C05.fsa	Bxpc-3	STR Profile 2-human		

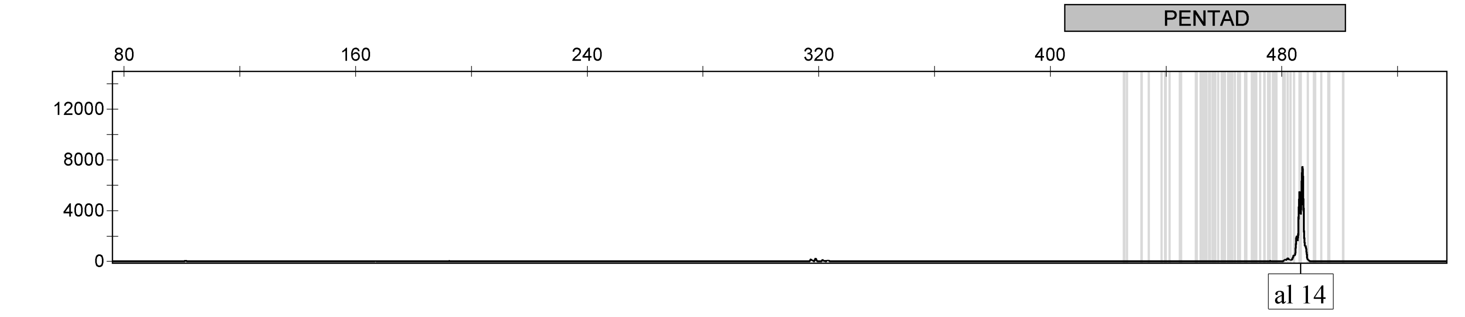

Sample File	Sample Name	Panel	OS	SQ
51_C07.fsa	Bxpc-3	STR Profile 3-human		

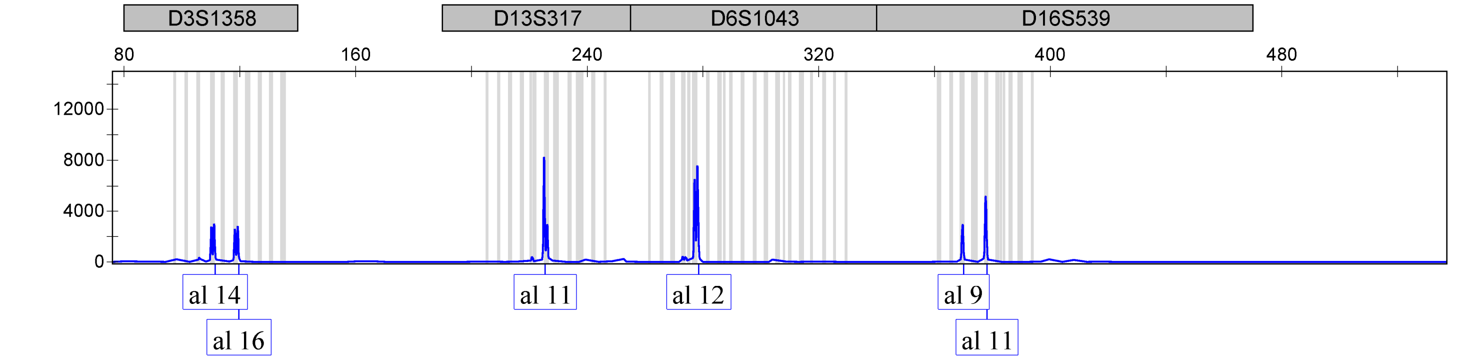

Sample File	Sample Name	Panel	OS	SQ
51_C07.fsa	Bxpc-3	STR Profile 3-human		

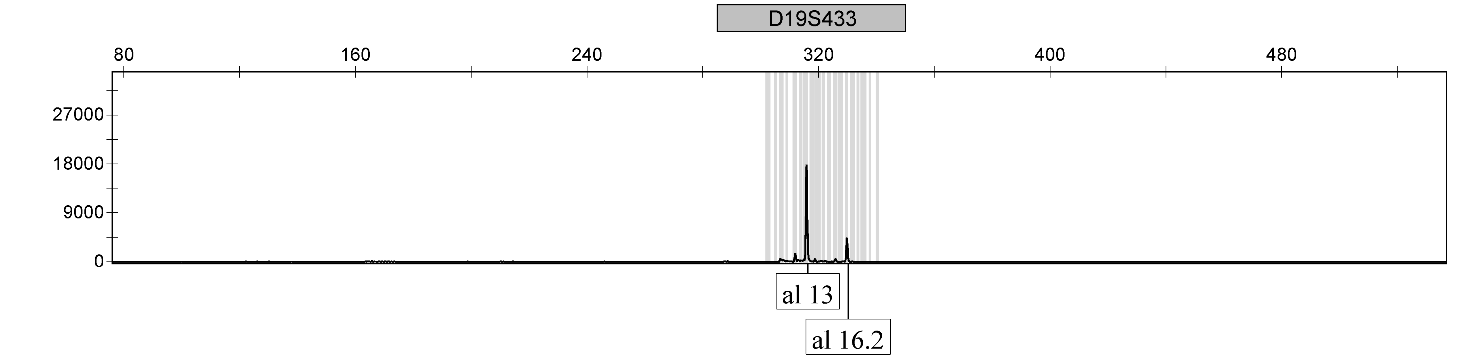

Sample File	Sample Name	Panel	OS	SQ
67_C09.fsa	Bxpc-3	STR Profile 4-human		

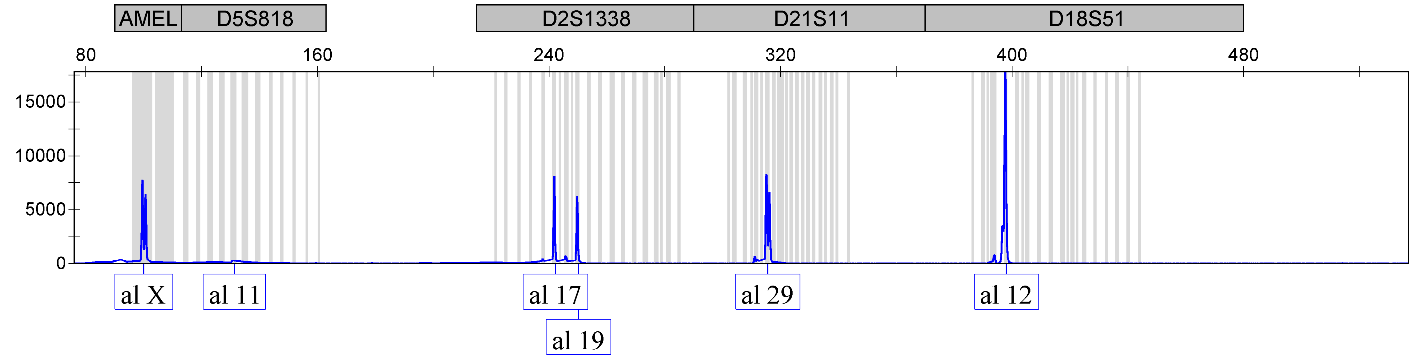

67_C09.fsa	Bxpc-3	STR Profile 4-human		

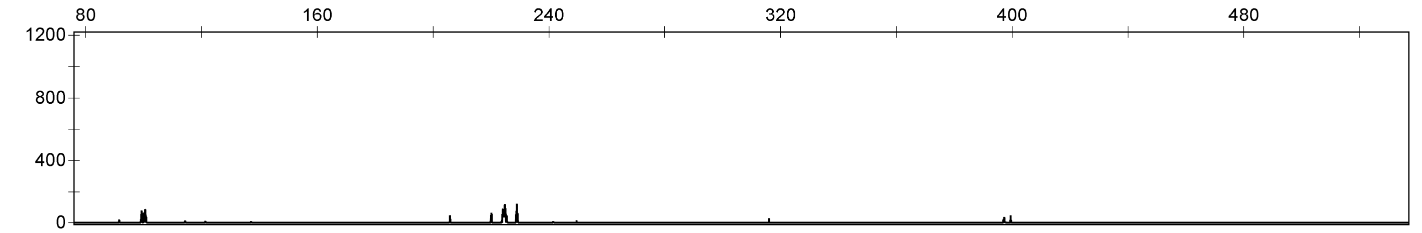

Supplement: Supplementary file 15 — BXPC-3-STR [file 41419_2023_5715_MOESM15_ESM.pdf]
