# Supplementary material for: METTL3 enhances pancreatic ductal adenocarcinoma progression and gemcitabine resistance through modifying DDX23 mRNA N6 adenosine methylation
Source: Cell Death Dis. 2023 Mar 28;14(3):221. doi: 10.1038/s41419-023-05715-1 (PMC10050319; doi:10.1038/s41419-023-05715-1)

细胞遗传质量鉴定检测

Cell Line Authentication Service

STR 基因型检测报告

样 品 信 息

样品编号：

客户样本编号	公司编号
Panc-1	20171113-01

样品数量： 1

样品性状： 细胞系

检测项目： STR

送检单位： 富衡

检测方法：用 Axygen 的基因组抽提试剂盒提取 DNA，采用 20- STR 扩增方案扩增，在 ABI 3730XL 型遗传分析仪上对 STR 位点和性别基因 Amelogenin 进行检测。

检 测 结 果

(一) 检验基本情况

	多等位基因	匹配细胞系	细胞库	EV 值	匹配说明
20171113-01	无	Panc-1	ATCC	1.00	完全匹配

样本基因型检验结果

- 多等位基因指三等位及以上基因现象。
- 本次检测各细胞分型结果良好。
- **各样本描述**
- 20171113-01：该株细胞 DNA 分型在细胞系检索中找到**完全匹配**的细胞系，ATCC 数据库显示细胞名为 **PANC-1**，细胞号对应 **CRL-1469**。本次检测在该细胞系中**没有发现多等位基因**。

备注：待测细胞系与收录于 ATCC, DSMZ, JCRB 和 RIKEN 数据库的细胞系 STR 数据进行比对，未收录于以上细胞库的细胞系将无法匹配。

（三）样本分型结果

**细胞** 20171113-01 **的 STR 位点和 Amelogenin 位点的基因分型结果**						
Loci	送检细胞 STR 信息			细胞库细胞 STR 信息		
	送检细胞名：Panc-1			细胞库细胞名：panc-1		
	Allele1	Allele2	Allele3	Allele1	Allele2	Allele3
D5S818	13	13		13	13	
D13S317	11	11		11	11	

D7S820	8	10		8	10	
D16S539	11	11		11	11	
VWA	15	15		15	15	
TH01	7	8		7	8	
AMEL	X	X		X	X	
TPOX	8	11		8	11	
CSF1PO	10	12		10	12	
D12S391	22	22				
FGA	21	21				
D2S1338	23	24				
D21S11	28	28				
D18S51	12	12				
D8S1179	14	15				
D3S1358	17	17				
D6S1043	11	12				
PENTAE	7	14				
D19S433	11	16				
PENTAD	14	14				

其他说明

(一) 分型方案及位点分布

	方案 1	方案 2	方案 3	方案 4

1	D3S1358	D8S1179	D19S433	AMEL
2	VWA	D21S11	TH01	D5S818
3	D7S820	D16S539	D13S317	D12S391
4	CSF1PO	D2S1338	TPOX	FGA
5	PENTAE	PENTAD	D18S51	
6			D6S1043	

实验方案及位点

(二) STR 数据库比对

本公司采用 DSMZ tools 进行细胞系比对，其中包含来自于 ATCC, DSMZ, JCRB 和 RIKEN 数据库的 2455 个细胞系 STR 数据。如果待检测细胞未收录于以上细胞库或这是自行建立的新细胞系将无法进行比对，用户需根据细胞分型结果自行与其他数据库进行比对。

签发日期：2017 年 12 月 1 日

GeneMapper 4.0

CellLineAuthentication-1122

Sample File	Sample Name	Panel	SQI	OS	SQ
73_A10_CellLineAuthentication-1-1127.FSA	panc-1	STR Profile 1-human			

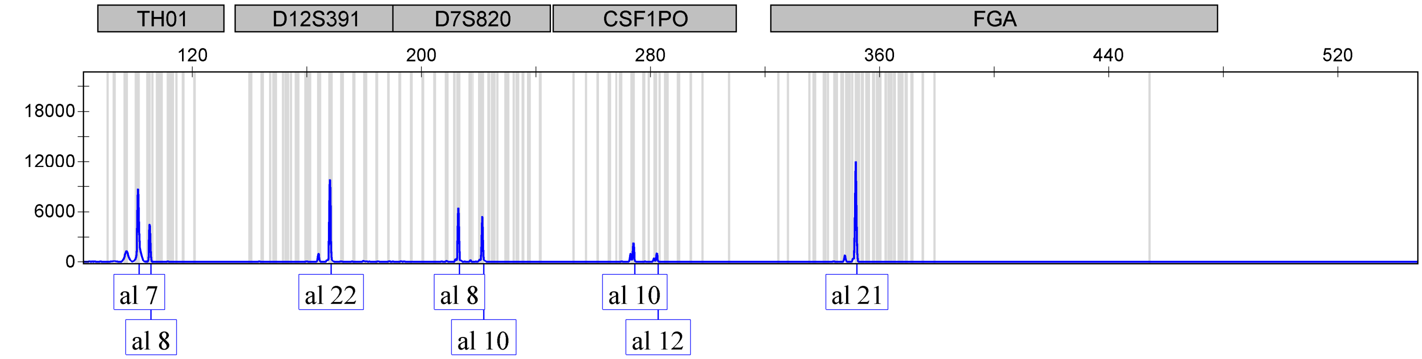

Sample File	Sample Name	Panel	SQI	OS	SQ
73_A10_CellLineAuthentication-1-1127.FSA	panc-1	STR Profile 1-human			

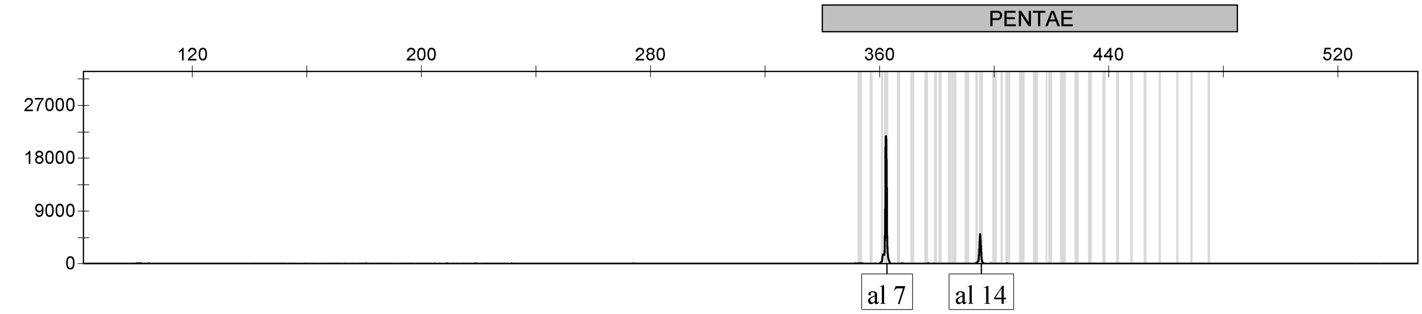

Sample File	Sample Name	Panel	SQI	OS	SQ
01_A01_CellLineAuthentication-2-1127.FSA	panc-1	STR Profile 2-human			

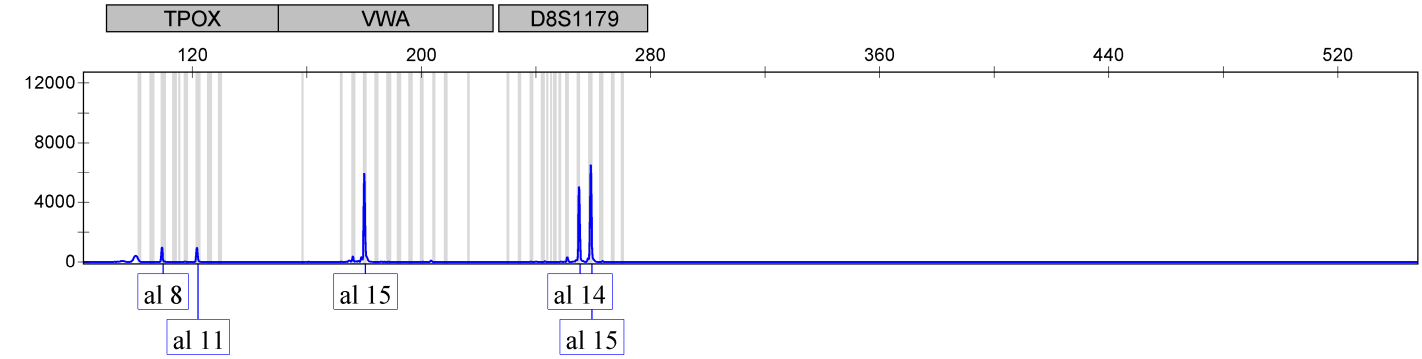

Sample File	Sample Name	Panel	SQI	OS	SQ
01_A01_CellLineAuthentication-2-1127.FSA	panc-1	STR Profile 2-human			

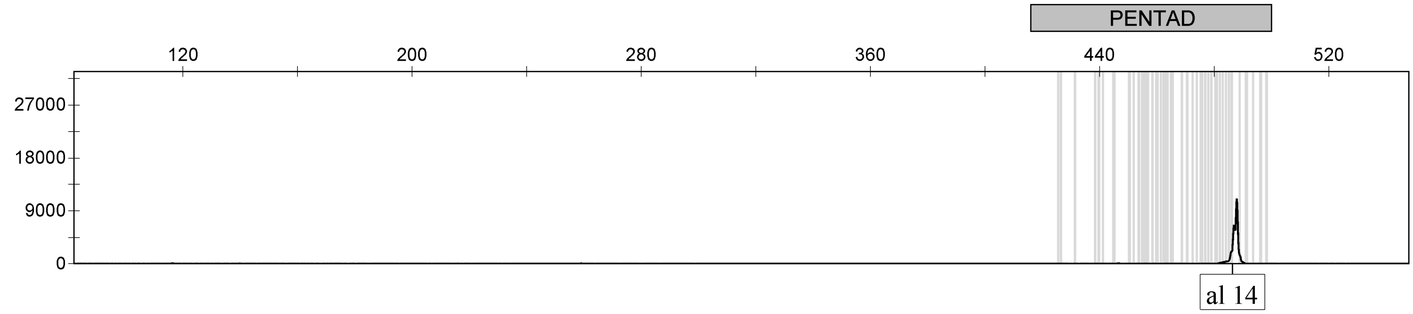

Sample File	Sample Name	Panel	SQI	OS	SQ
41_A06_CellLineAuthentication-2-1127.FSA	panc-1	STR Profile 3-human			

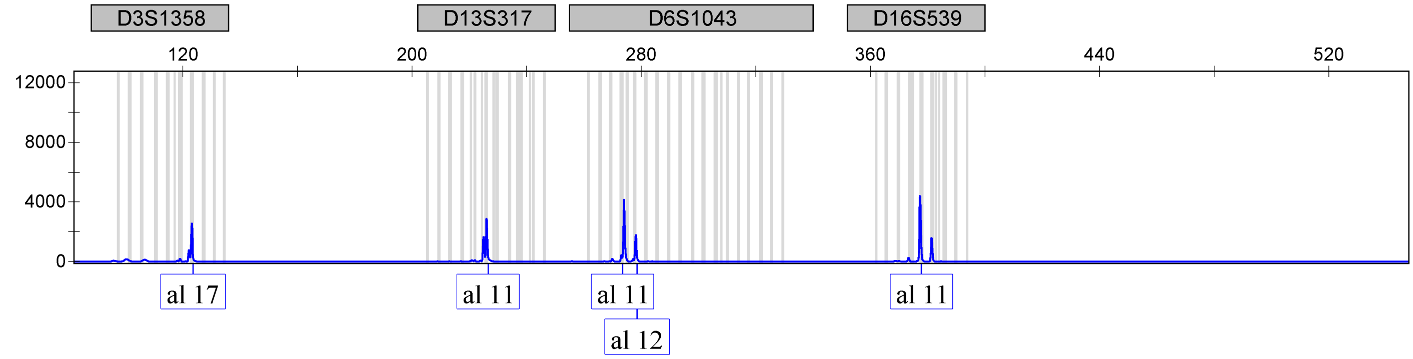

Sample File	Sample Name	Panel	SQI	OS	SQ
41_A06_CellLineAuthentication-2-1127.FSA	panc-1	STR Profile 3-human			

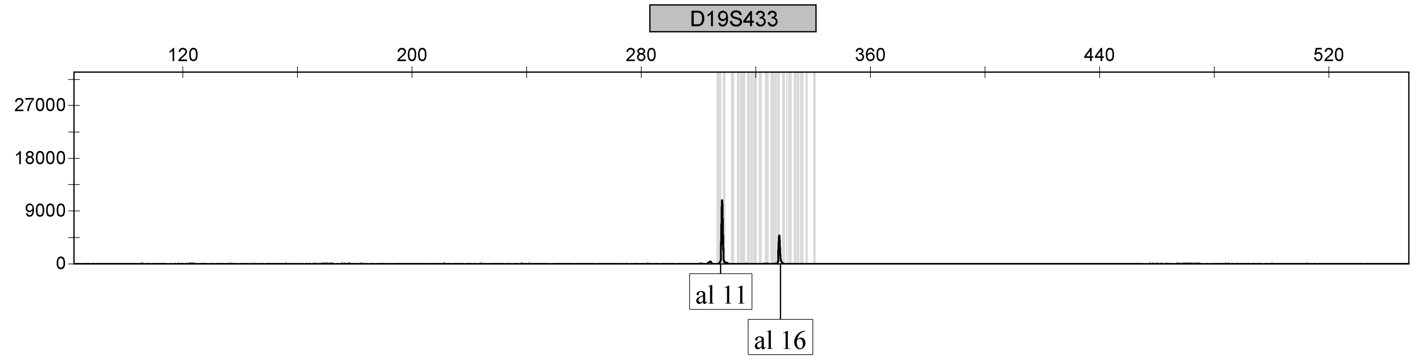

CellLineAuthentication-1122

Sample File	Sample Name	Panel	SQI	OS	SQ
01 A01 XiBaoJianDing1122Pi-2(1-5)-1129.FSA	panc-1	STR Profile 4-human			

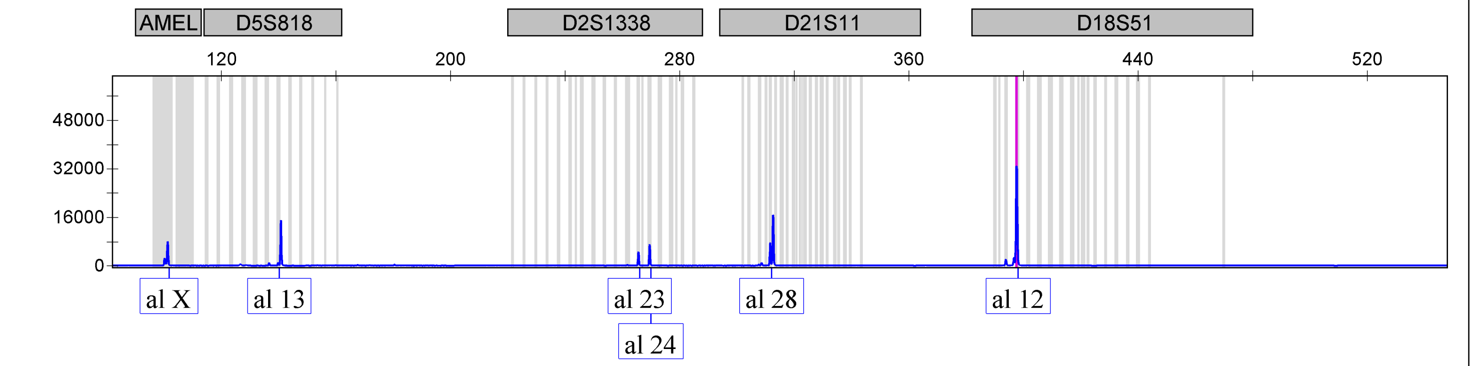

01 A01 XiBaoJianDing1122Pi-2(1-5)-1129.FSA	panc-1	STR Profile 4-human			

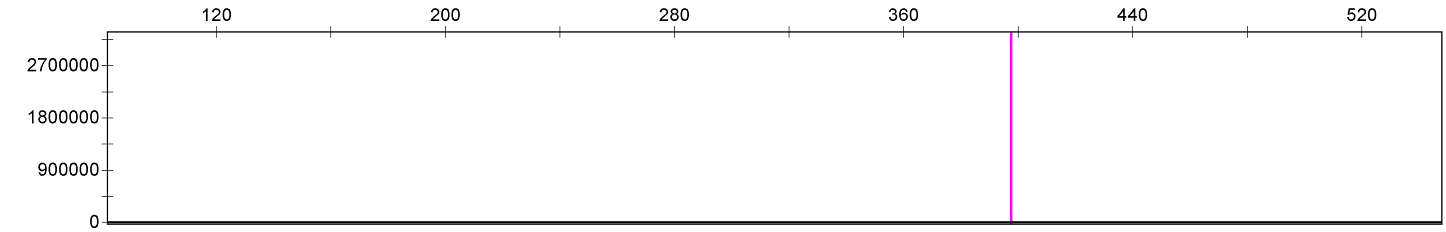

Supplement: Supplementary file 16 — panc-1-STR [file 41419_2023_5715_MOESM16_ESM.pdf]
