# Supplementary material for: METTL3 enhances pancreatic ductal adenocarcinoma progression and gemcitabine resistance through modifying DDX23 mRNA N6 adenosine methylation
Source: Cell Death Dis. 2023 Mar 28;14(3):221. doi: 10.1038/s41419-023-05715-1 (PMC10050319; doi:10.1038/s41419-023-05715-1)

细胞遗传质量鉴定检测

Cell Line Authentication Service

STR 基因型检测报告

样 品 信 息

样品编号：

客户样本编号	公司编号
SW1990	20170831-02

样品数量：1

样品性状：细胞系

检测项目：STR

送检单位：富衡生物

检测方法：用 Axygen 的基因组抽提试剂盒提取 DNA，采用 20- STR 扩增方案扩增，在 ABI 3730XL 型遗传分析仪上对 STR 位点和性别基因 Amelogenin 进行检测。

检 测 结 果

(一) 检验基本情况

	多等位基因	匹配细胞系	细胞库	EV 值	匹配说明
20170831-02	无	SW 1990 [SW-1990, SW1990]	DSMZ	1.00	完全匹配

样本基因型检验结果

- 多等位基因指三等位及以上基因现象。
- 本次检测各细胞分型结果良好。

(二) 各样本描述

- 20170831-01：该株细胞 DNA 分型在细胞系检索中找到**完全匹配**的细胞系，DSMZ 数据库显示细胞名为 **SW 1990 [SW-1990, SW1990]**，细胞号对应 **CRL-2172** 。本次检测在该细胞系中**没有发现多等位基因**。

  备注：待测细胞系与收录于 ATCC, DSMZ, JCRB 和 RIKEN 数据库的细胞系 STR 数据进行比对，未收录于以上细胞库的细胞系将无法匹配。

（三）样本分型结果

细胞 20170831-01 的 STR 位点和 Amelogenin 位点的基因分型结果						
Loci	送检细胞 STR 信息			细胞库细胞 STR 信息		
	送检细胞名：SW1990			细胞库细胞名：SW 1990 [SW-1990, SW1990]		
	Allele1	Allele2	Allele3	Allele1	Allele2	Allele3
D5S818	12	13		12	13	
D13S317	8	12		8	12	
D7S820	9	10		9	10	
D16S539	13	13		13	13	
VWA	17	17		17	17	
TH01	9.3	9.3		9.3	9.3	
AMEL	X	X		X	X	
TPOX	8	9		8	9	
CSF1PO	10	12		10	12	
D12S391	17	17				
FGA	26	26				
D2S1338	23	23				
D21S11	31	31				
D18S51	13	13				
D8S1179	11	14				
D3S1358	16	16				
D6S1043	11	19				
PENTAE	5	7				
D19S433	13	15				
PENTAD	11	11				

其 他 说 明

(一)　分型方案及位点分布

	方案 1	方案 2	方案 3	方案 4
1	TH01	TPOX	D3S1358	AMEL
2	D12S391	VWA	D13S317	D5S818
3	D7S820	D8S1179	D6S1043	D2S1338
4	CSF1PO	PENTAD	D16S539	D21S11
5	FGA		D19S433	D18S51
6	PENTAE			

实验方案及位点

(二)　STR 数据库比对

本公司采用 DSMZ tools 进行细胞系比对，其中包含来自于 ATCC, DSMZ, JCRB 和 RIKEN 数据库的 2455 个细胞系 STR 数据。如果待检测细胞未收录于以上细胞库或这是自行建立的新细胞系将无法进行比对，用户需根据细胞分型结果自行与其他数据库进行比对。

签发日期：2017 年 09 月 13 日

Sample File	Sample Name	Panel	OS	SQ
66_B09_CellLineAuthentication-1-0906.fsa	SW1990	STR Profile 1-human		

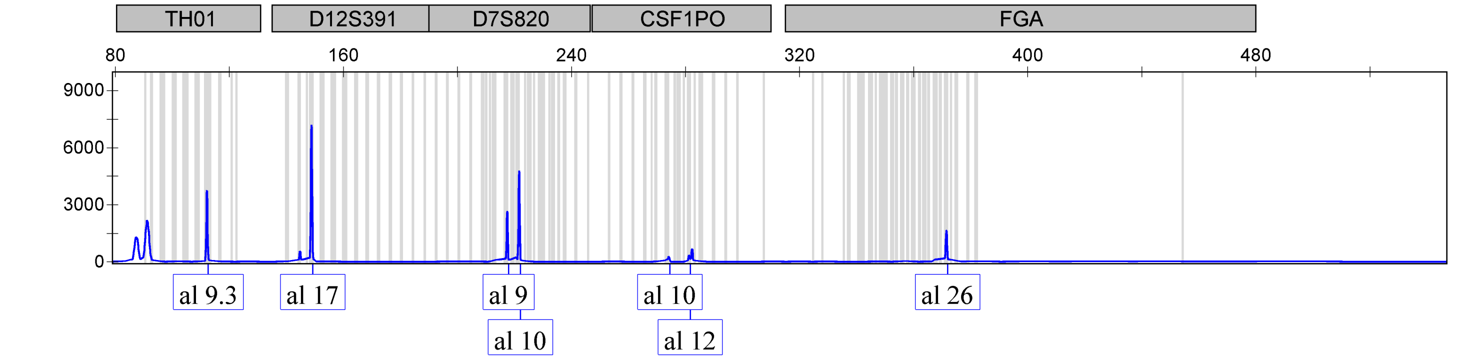

Sample File	Sample Name	Panel	OS	SQ
66_B09_CellLineAuthentication-1-0906.fsa	SW1990	STR Profile 1-human		

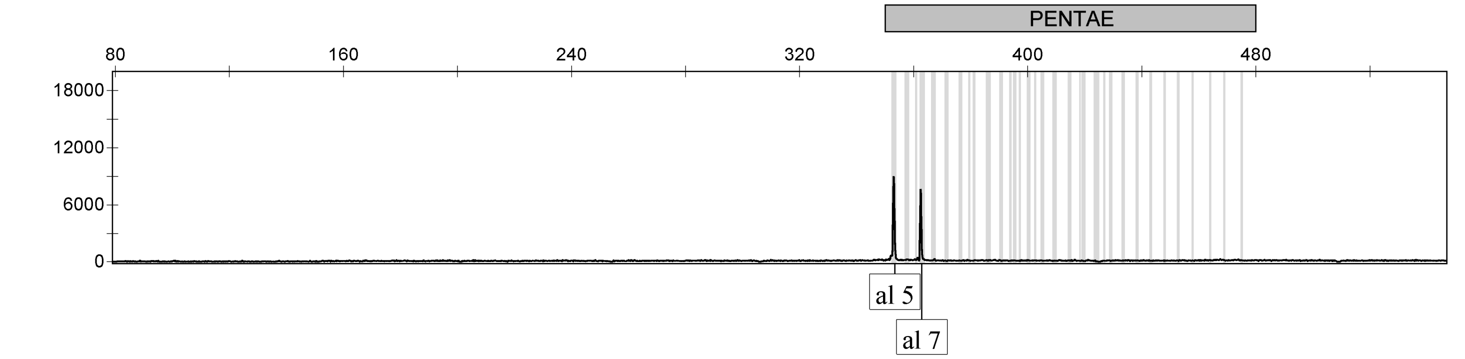

Sample File	Sample Name	Panel	OS	SQ
42_B06_CellLineAuthentication-2-0906.fsa	SW1990	STR Profile 2-human		

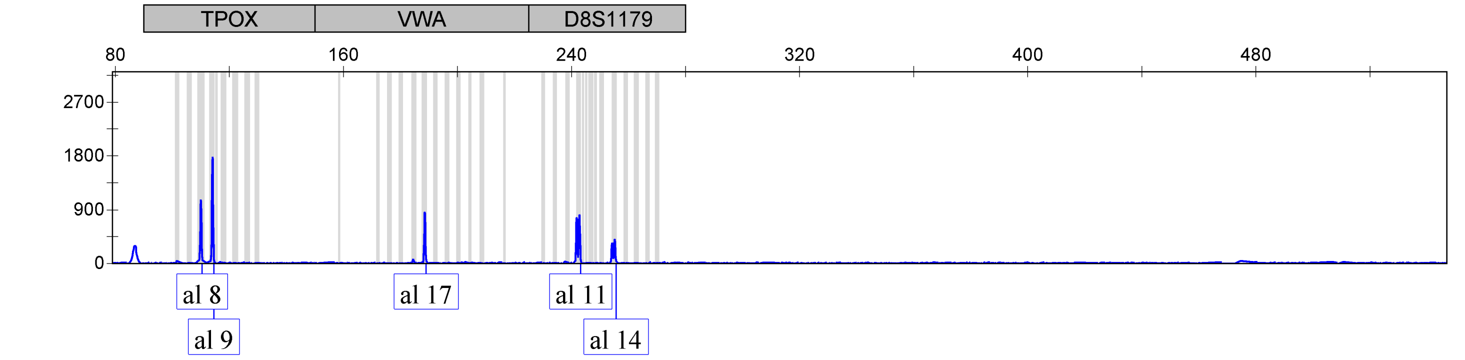

Supplement: Supplementary file 17 — sw1990-STR [file 41419_2023_5715_MOESM17_ESM.pdf]
